# Digital dashboards visualizing public health data: a systematic review

**DOI:** 10.3389/fpubh.2023.999958

**Published:** 2023-05-04

**Authors:** Annett Schulze, Fabian Brand, Johanna Geppert, Gaby-Fleur Böl

**Affiliations:** Study Centre for Social Science Research, Department Risk Communication, German Federal Institute for Risk Assessment, Berlin, Germany

**Keywords:** visualization, risk information, health literacy, information needs, representations, dashboard

## Abstract

**Introduction:**

Public health is not only threatened by diseases, pandemics, or epidemics. It is also challenged by deficits in the communication of health information. The current COVID-19 pandemic demonstrates that impressively. One way to deliver scientific data such as epidemiological findings and forecasts on disease spread are dashboards. Considering the current relevance of dashboards for public risk and crisis communication, this systematic review examines the state of research on dashboards in the context of public health risks and diseases.

**Method:**

Nine electronic databases where searched for peer-reviewed journal articles and conference proceedings. Included articles (*n* = 65) were screened and assessed by three independent reviewers. Through a methodological informed differentiation between descriptive studies and user studies, the review also assessed the quality of included user studies (*n* = 18) by use of the Mixed Methods Appraisal Tool (MMAT).

**Results:**

65 articles were assessed in regards to the public health issues addressed by the respective dashboards, as well as the data sources, functions and information visualizations employed by the different dashboards. Furthermore, the literature review sheds light on public health challenges and objectives and analyzes the extent to which user needs play a role in the development and evaluation of a dashboard. Overall, the literature review shows that studies that do not only describe the construction of a specific dashboard, but also evaluate its content in terms of different risk communication models or constructs (e.g., risk perception or health literacy) are comparatively rare. Furthermore, while some of the studies evaluate usability and corresponding metrics from the perspective of potential users, many of the studies are limited to a purely functionalistic evaluation of the dashboard by the respective development teams.

**Conclusion:**

The results suggest that applied research on public health intervention tools like dashboards would gain in complexity through a theory-based integration of user-specific risk information needs.

**Systematic review registration:**

https://www.crd.york.ac.uk/prospero/display_record.php?RecordID=200178, identifier: CRD42020200178.

## 1. Introduction: monitoring public health

The current COVID-19 pandemic poses immense challenges for nation-states and civil society alike. Not only does the current situation severely restrict public and private life, but also affects governmental agencies which are constantly confronted with dynamic decision-making situations. Both private individuals and decision-makers are carefully observing developments and using different types of media and formats to make sense of the current crisis as well as finding appropriate ways to communicate data and messages (1). Quality media such as public service broadcasting in Germany use figures from universities or from national and international health organizations such as the Robert Koch-Institute (RKI) or the World Health Organization (WHO) in their reporting. The findings and forecasts on the spread of the virus are increasingly presented in so-called dashboards (2) i.e., through a specific type of visualization “of a consolidated set of data for a certain purpose” (3), using a combination of numerical, temporal, geographical, and diagrammatic forms of presentation.

These dashboards capture the extent of the outbreak by visualizing cases, hospitalizations, deaths, vaccination rates etc. and allow to track the outbreak from a regional up to a global scope. They can be used to gain a quick overview, allow specific analysis and facilitate decision-making. Thereby, surveillance activities provide an instrument to prevent diseases, reduce morbidity and mortality, and promote health—objectives that define public health (4).

Worldwide, the globalization and the dissolution of national boundaries for diseases, disease spread, pollution, or environmental catastrophes foster the emergence of public health surveillance infrastructures (5) including a wide range of mobile health tools (6). With the expanding digitization, data-driven developments become more important for the assessment and surveillance of public health issues (7, 8). In the context of infectious disease surveillance, for example, dashboards are often the focus of scientific interest as a tool for visualizing epidemic data (9, 10). The focus of these studies is on increasing the efficiency of surveillance systems by identifying potential gaps—ranging from technical improvements over data quality to modeling these data. Additionally, the COVID-19 pandemic has shown, that not only epidemiologists, statisticians or data modelers are interested in near real-time COVID-19 data (11), but also the general public seeks for information about the spread of the virus (12, 13).

Therefore, the evaluation of an online communication format such as a dashboard is important with regard to many different aspects. Through a meta literature review, we were able to crystallize a not necessarily exhaustive but nonetheless comprehensive list of four different aspects that are important to consider in dashboard research. Major aspects mentioned in the literature here were (a) how public health data is visualized (14, 15), (b) the modes of communication used (16), (c) how the visualized data can be understood, is read and filled with meaning by various subpopulations (17, 18), and (d) how effective different (communication) formats are (16, 17, 19). At the same time, the large amount of data that can be provided via dashboards, as well as their scientific nature, pose various challenges to users—whether in understanding, processing or contextualizing the information (13, 20). Accordingly, there is a need for research on the needs of users.

Until 2020 and to the best of our knowledge, no systematic review on public health dashboards existed. Only two other reviews have appeared in this context by now (June 2022). A literature review provides insights into the technological advances of dashboards (21). One dashboard review sheds light on design modes of U.S. COVID-19 State Government Public Dashboards (15).

Therefore and from a communication science perspective, we investigate scientific studies on dashboards as a form of diagrammatic images in science communication covering public health issues—from non-communicable diseases (e.g., diabetes), communicable diseases (e.g., Ebola) and natural disasters (e.g., floods) to addictive disorders and related health risks such as drug abuse (22) or obesity (23). These behavioral risk factors have a public health impact as they can cause non-communicable diseases. We are particularly interested in whether empirical analysis will provide indications for a more effective visualization of scientific data, e.g., by drawing on cognitive and affective factors to process visual information. Thus, this systematic review aims to assess the state of research on dashboards, that are utilized in a public health context and provide information on divergent public health phenomena such as risks, pandemics, infections or health crises, with a focus on the methods of gathering and presenting public health information as well as the methodological approaches used to develop or evaluate the dashboards.

## 2. Methods

Our systematic literature review followed the steps, comprehensively described by Xiao and Watson (24): (1) formulating the research problem, (2) developing and validating the review protocol, (3) searching the literature, (4) screening for inclusion, (5) assessing quality, (6) extracting data, (7) analyzing and synthesizing data, and (8) reporting the findings.

### 2.1. Formulating the research problem

Research on the effective visualization of scientific data through dashboards from a communication science perspective is scarce. This literature review is therefore devoted to two distinctive objectives, which in turn are structured by a total of three research questions (RQs). First, it aims to offer an overview of different dashboards described in the scientific literature as relevant to the field of public health, thereby encompassing elements of a scoping review (RQ 1 & RQ 2). Second, it pursues to gain insights into the needs and demands of different user groups while engaging with a public health dashboard (RQ 3). Answers to the last research question are expected to be gained exclusively from those studies that have conducted a user study, assessing their specific needs and demands. Thus, the review needs to further differentiate between user studies and mere descriptive studies (see Section 2.5). In that sense, the derived research questions have been defined as follows:

- RQ 1: Which dashboards that are thematically related to the field of public health have been examined in the scientific, peer-reviewed literature and what is known about them? In particular:

◦ RQ 1.1: Which areas relevant to public health—such as diseases, risks or crises—are covered by these dashboards?
◦ RQ 1.2: From which sources do these dashboards retrieve their data?
◦ RQ 1.3: What information (data or indicators) is visualized through these dashboards?
◦ RQ 1.4: Which graphical representations are used to visualize the data or indicators in these dashboards?
◦ RQ 1.5: Which functions do these dashboards offer besides the pure visualization of information?

- RQ 2: Which challenges and objectives are addressed in the sampled articles (a) in regards to the consolidation of public health and (b) in regards to the use of dashboards in that specific context?

◦ RQ 2.1a: Which public health challenges do the sampled articles address?
◦ RQ 2.1b: What public health objectives are they pursuing?
◦ RQ 2.2a: What specific technological or administrative challenges are associated with the use of dashboards in public health?
◦ RQ 2.2b: What are the specific technological or administrative objectives associated with the use of dashboards in public health?

- RQ 3: Which information needs can be identified in the assessed user studies regarding the engagement with public health dashboards?

### 2.2. Developing and validating the review protocol

Before the systematic search was carried out, we conducted a cursory review and pre-review mapping of relevant articles on the use of dashboards in public health settings. These articles were identified through quick-scan searches in various databases such as Scopus or Google Scholar. A loose combination of search words (such as “public health dashboard”, “evaluation”, or “perception”) was used in order to obtain an overview of the body of literature on dashboard research and to identify possible keywords for the definition of viable search strings.

### 2.3. Searching the literature—identifying relevant articles

After formulating the research questions, validating, and publishing our research protocol on PROSPERO (CRD42020200178), two different search strings were conceptualized in order to retrieve relevant articles. Using Boolean operators “AND”, “OR”, “NOT”, the first search string combined different user-centered (e.g., “literacy” or “knowledge”) as well as visualization-centered (e.g., “graph” or “multimodal”) keywords with the search term “dashboard” and different areas of public health (e.g., “epidemiology”). The focus on these categories is intended to limit the broad field of dashboard research to those articles that specifically relate to the field of public health and potentially address the question of user preferences and design considerations. Due to the increasing and striking relevance of dashboards in the context of the current COVID-19 pandemic [for a critical discussion see Everts (25)], we further defined an additional search string, covering a spectrum of recently published articles on COVID-19-relevant dashboards.

To conduct the review, multi-disciplinary databases such as Scopus, Web of Science, technical-oriented databases like IEEE Xplore and ACM Digital Library and databases from different disciplinary fields such as communication sciences (Communication Abstracts, Communication & Mass Media Complete) or psychology (PsycArticles, PsycInfo) were selected. We included Open Gray as an additional database to identify further relevant papers. Through this range of databases, it is assumed that a wide range of literature on public health dashboards is covered, as, for example, Scopus also includes records from the MEDLINE and EMBASE databases.

### 2.4. Screening for inclusion

**Figure 1:**
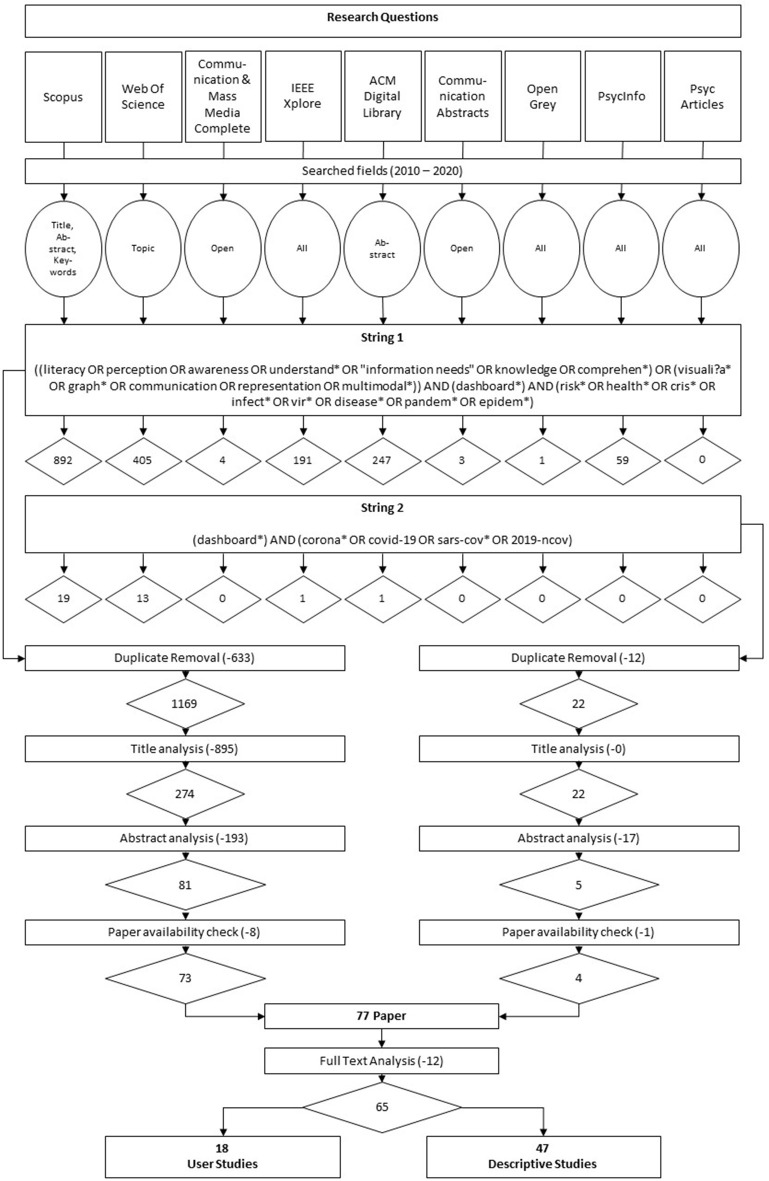
Research questions and visualization of the literature search process including search strings and number of retrieved and assessed publication.

Before running both search strings in the mentioned academic databases, several inclusion and exclusion criteria were defined in order to evaluate identified papers for further consideration in the literature review (eligibility assessment). These criteria are presented in Appendix A. Figure 1 illustrates the complete search process.

After retrieving a total of 1,836 papers by running both search strings in the aforementioned nine academic databases (see Section 2.3), an automated duplicate removal, supplemented by a subsequent hand search for duplicates, reduced our sample to a total of 1,191 papers.

These remaining 1,191 papers went through different selection stages. To test for interrater reliability two researchers randomly selected 100 papers from our sample and assessed their titles for further selection based on the previously defined inclusion and exclusion criteria (see Appendix A). Belur et al. (26) report several methods for calculating interrater reliability, including Cohen's κ, where a score of 1 indicates perfect agreement and a score of 0 equates agreement totally due to chance. By comparing individual ratings, we finally calculated a Cohen's κ of 0.78—implying, according to Landis and Koch (27), substantial agreement.

Our review applies a titles-first then abstracts screening strategy, which was already recommended by Mateen et al. (28) based on an empirical comparison of different screening methods, as a titles-first strategy guarantees an “accurate, less time-consuming process that does not compromise the quality of the final review”. In accordance with a previously defined code book, supplementing our defined inclusion and exclusion criteria (see Appendix A), all 1,191 identified papers were assessed for eligibility based on their titles. This procedure left us with 296 remaining papers of which all titles and abstracts were read and assessed for eligibility in accordance with the above mentioned inclusion and exclusion criteria. Critical or unclear cases were deferred for further review by all researchers involved. Finally, discrepancies or disagreements concerning the eligibility assessment were solved by discussion and consensus-based decision-making. The review of the remaining abstracts left us with a total of 86 papers. However, nine more papers had to be further excluded from the study either because they were not available or could not be acquired. After a thorough reading of identified and potentially relevant full-text articles as well as a consequent reapplication of the defined inclusion and exclusion criteria, we finally selected 65 articles for our final literature review.

### 2.5. Assessing quality

**Figure 2:**
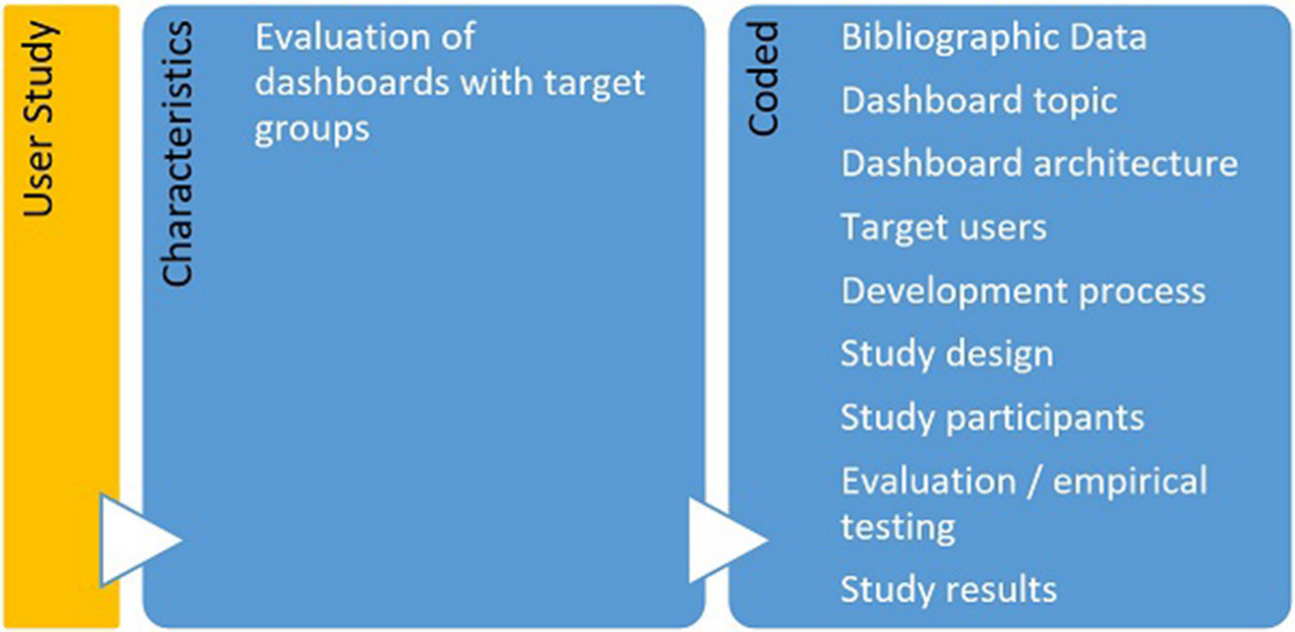
Characteristics and associated codes for user studies.

**Figure 3:**
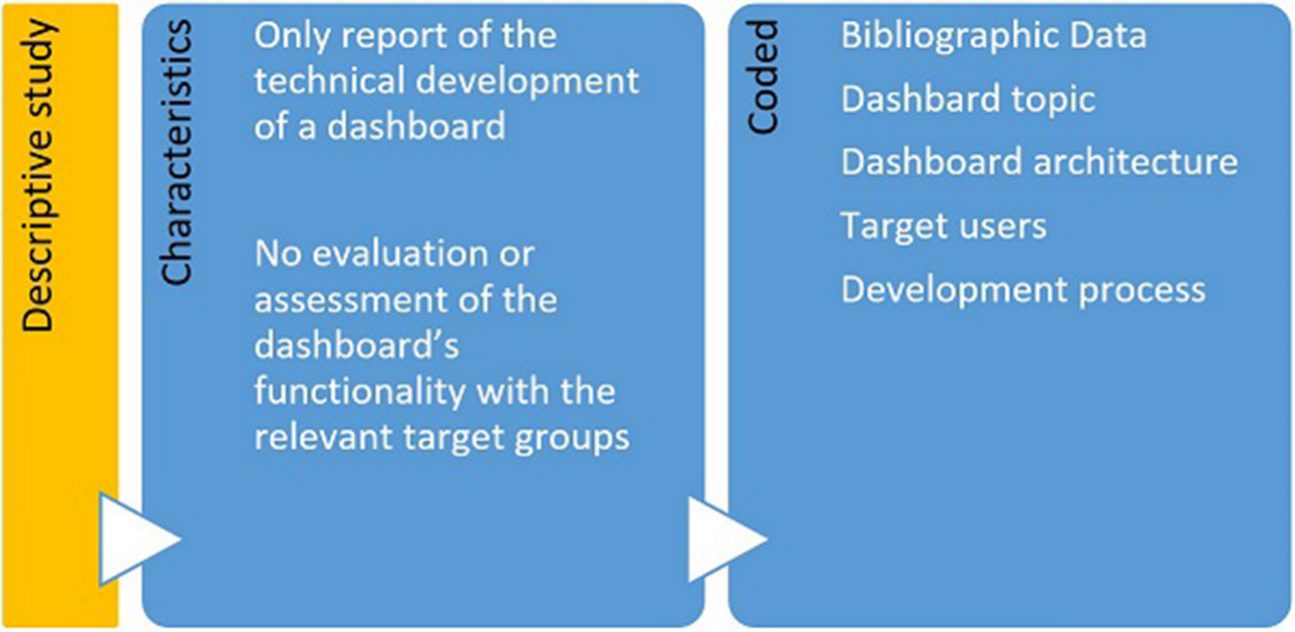
Characteristics and associated codes for descriptive studies.

In order to adequately assess the quality of identified studies, we developed a scheme to differentiate the selected 65 articles according to their empirical focus (see Figures 2, 3). Studies that had executed a user study (*n* = 18), meaning an empirical assessment of a focal dashboard through different user groups, were considered for further quality assessment by means of the Mixed Methods Appraisal Tool (MMAT) which was specifically developed for critically appraising the quality of different study designs in systematic mixed studies reviews (29).

The MMAT provides the possibility of assigning ratings in order to record the quality of the included studies by using descriptors such as (^*^) or (%). The final quality rating is determined by the summarized total number of “yes” items assigned to the respective study category (e.g., qualitative studies). For mixed-methods studies, the developers of the MMAT state that “the overall quality of a combination cannot exceed the quality of its weakest component” (30). Since there are 15 criteria to rate for mixed-methods studies (including the five items for the first applied method as well as five items for the second method employed in the respective articles), the overall score for these types of studies is based on the lowest score of all considered study components.

The remaining 47 articles focused either on the development of dashboards and their respective testing through various IT-related measures or on the pure description of a respective dashboard system and were classified as descriptive studies. They were considered relevant for answering the defined research questions as well and thus incorporated in the next step.

### 2.6. Extracting, analyzing, and synthesizing data

After performing a comprehensive quality assessment, all 65 articles were finally coded with MAXQDA according to the research questions, defined above. Both inductive and deductive coding was used. Three researchers were involved in the inter-coder process to achieve coding consistency (31, 32). Disagreements were debated until consent was reached. After the first tests for consistency, all papers were coded by two researchers. Whenever discrepancies arose, a third researcher was consulted. Every time a new code was added to the coding system, all papers that had already been coded were revised again. After initial coding and fine-tuning of respective coding categories, further fine coding was carried out, which formed the basis for the results reported below.

## 3. Results: answering the research questions

### 3.1. Public health dashboards in the scientific literature providing information on public health issues (RQ 1)

#### 3.1.1. Public health issues covered by dashboards (RQ 1.1)

**Figure 4:**
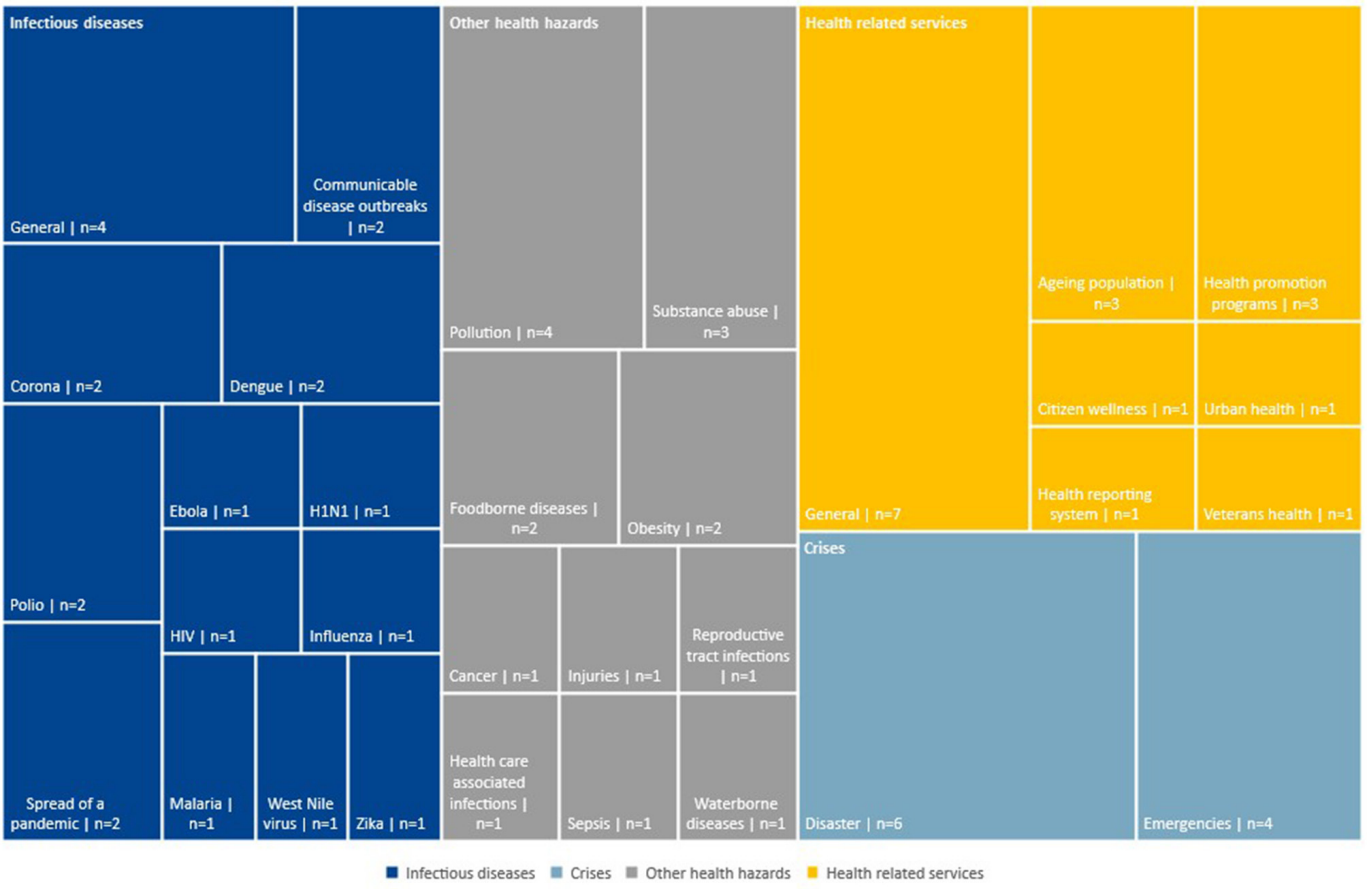
Subcategories of the four main dashboard topics.

In total 65 papers were included in our literature review. They cover topics from infectious diseases like Dengue (33), Ebola (34), or COVID-19 (35) (*n* = 21), crises caused by emergencies and disasters, such as floods [e.g., (36)] (*n* = 6) or other health hazards such as those caused by pollution (e.g., 37) (*n* = 4) (see Appendix B for raw data, Figure 4 on dashboard topics).

#### 3.1.2. Data sources used by dashboards (RQ 1.2)

Data displayed on the dashboards is derived from different sources like (a) governmental institutions (37) (*n* = 14), (b) health organizations like the World Health Organization and health care facilities (38) (*n* = 25) (c) national or local Research Organizations like the National Center for Health Statistics (39) (*n* = 6), (d) cities or communities (40) (*n* = 11), (e) news and journals (41) (*n* = 8), and (f) social media such as Twitter (42) (*n* = 8). Also, eleven papers report that (g) the users of the dashboard can be a source of information (43). Often dashboards derive their information from more than one source (see Appendix C). For example, Zheng et al. (44) created a dashboard to exchange critical information for the private and public sector in case of a crisis situation. The information is gathered from County Emergency Offices, company reports and messages as well as the news. Also, users can add further reports. Another dashboard tracking COVID-19 cases collects and displays data from a medical community online platform as well as Twitter and online news (35).

#### 3.1.3. Information (data or indicators) visualized through dashboards (RQ 1.3)

As stated above, the papers analyzed describe dashboards that deal with the visualization of data on, for example, diseases, crises and risks. Key indicators mentioned in different studies are (see Appendix D):

1. The number of reported cases (e.g., of a disease) or rates (e.g., death rates) (*n* = 15).
2. Health data including patient attributes (e.g., weight) and type of disease (e.g., HIV) (*n* = 43).
3. Social and environmental factors (e.g., education) (*n* = 7).
4. Environmental data (e.g., air pollution, temperature) (*n* = 15).
5. Demographics (e.g., age, gender) (*n* = 14).
6. Time (e.g., time of an event, variation in time) (*n* = 14).
7. Location (e.g., region or country) (*n* = 38).

#### 3.1.4. Graphical representations used to visualize data or indicators in dashboards (RQ 1.4)

**Figure 5:**
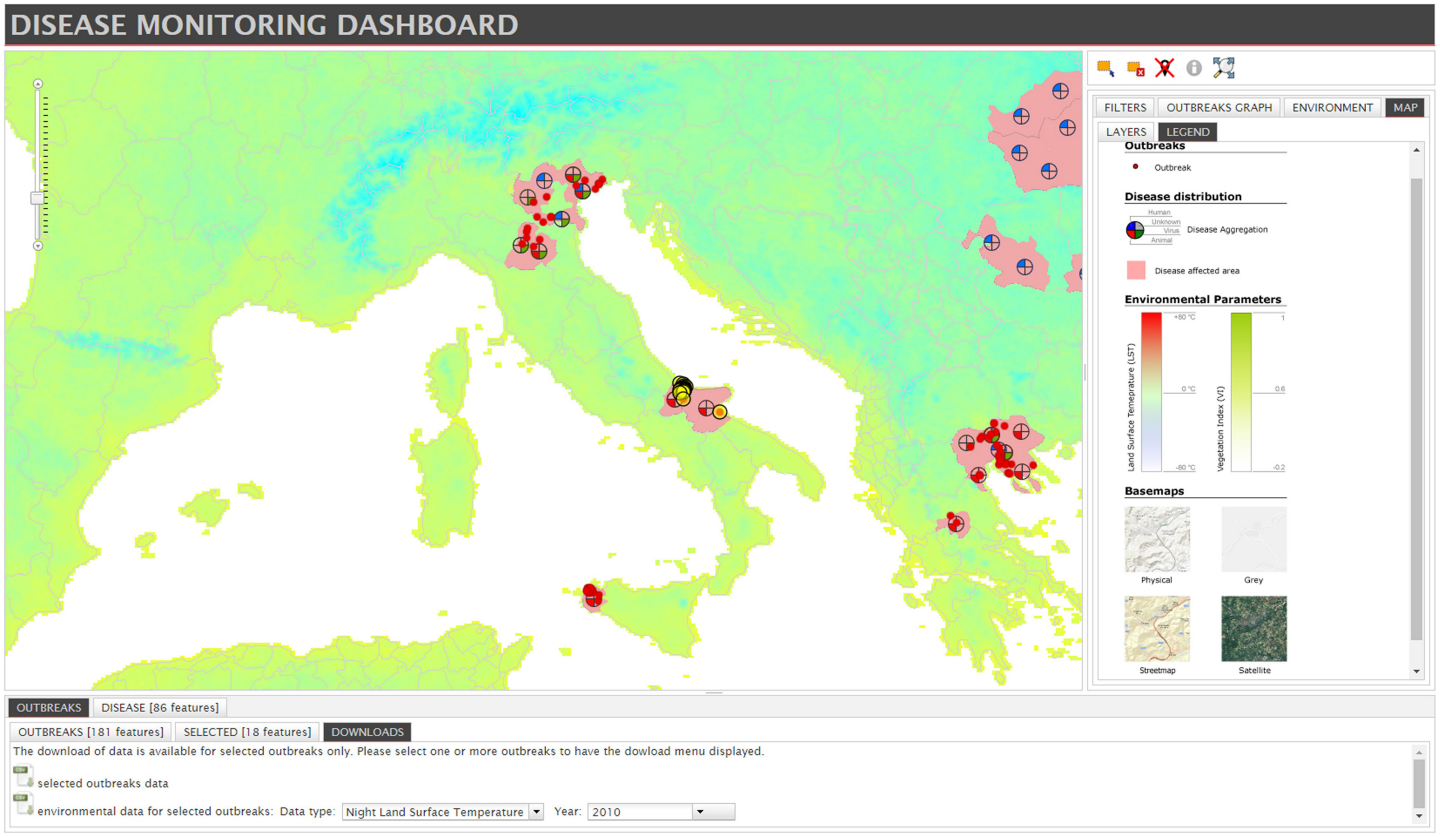
Example for a map with symbols, taken from “Disease Monitoring Dashboard” by Lara Savini et al. is licensed under CC BY 4.0 (38).

The visualization of data is one of the main goals of the dashboards. To do so, the dashboards mainly feature maps (see Figure 5), charts and tables. Forty dashboards reporting incidences of health hazards or the magnitude of a crisis caused e.g., by natural disasters, use maps to visualize the spread or effected areas (45). These are often further enhanced by symbols (38) (*n* = 5), icons (46) (*n* = 6) or pop-ups (47) (*n* = 11) that become visible when the users hover over the map.

**Figure 6:**
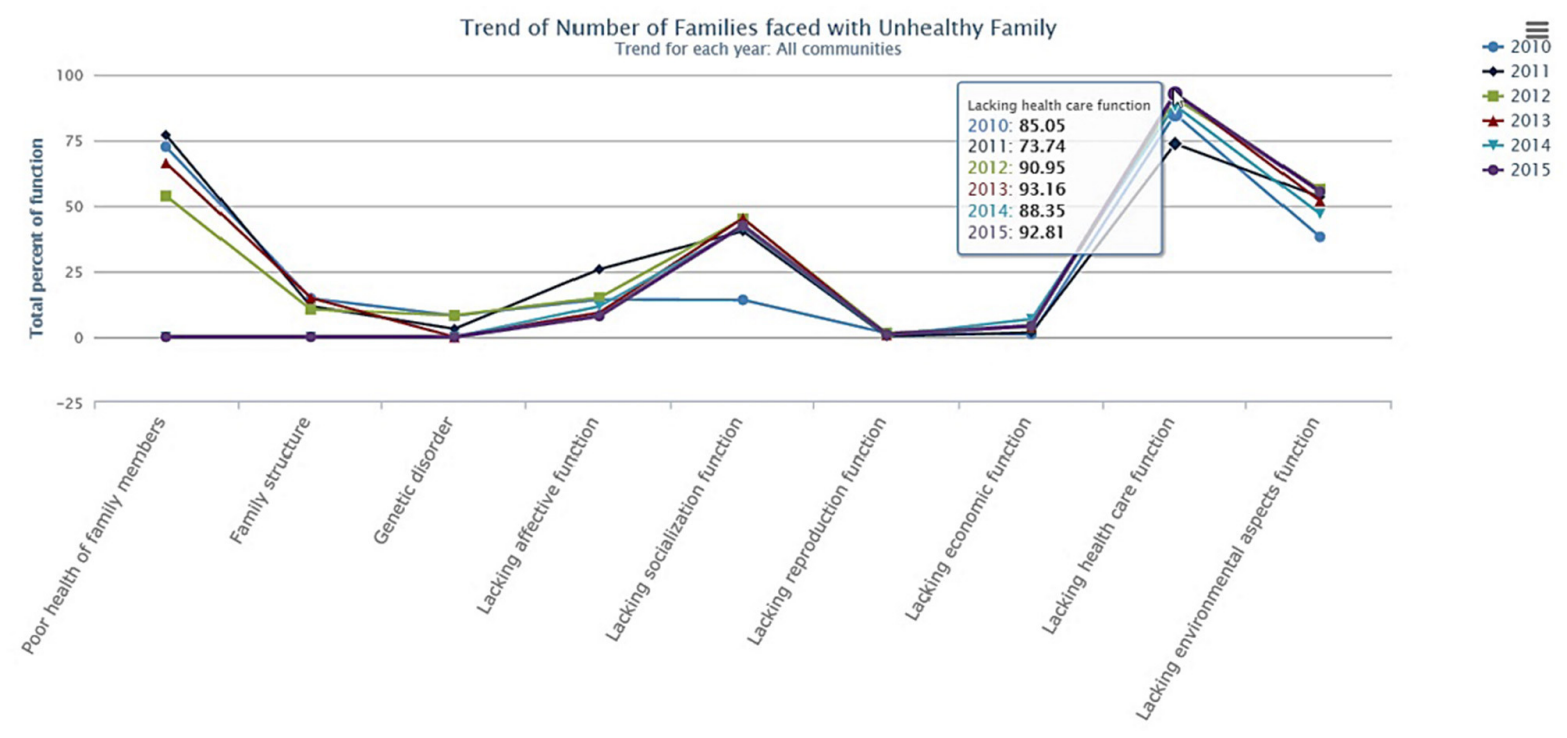
Example for a line graph, taken from “Trend of Number of Families faced with Unhealthy Family” by Puangrat Jinpon et al. is licensed under CC BY-NC-ND 4.0 (49).

Charts and graphs are used in different formats such as bar charts (48) (*n* = 24), pie charts (33) (*n* = 16), or line graphs (49) (*n* = 6; see Figure 6). All types of charts and graphs facilitate date visualization in general but it is not further explained how the developers of the dashboards decided which type of chart or graph they were going to use. Tables are used to display rankings, precise numbers and scores and to list different data on one aspect (50) (*n* = 21).

Besides the mentioned, common visualizations, four dashboards incorporate timelines aiming at a more holistic understanding of the situation and analyze events over a period of time (41, 51). Concannon et al. (47), for example, uses tree maps as they are preferred by the users of the dashboard and allow for more precise display of labels. Word clouds are primarily used to visualize social media data such as keywords from Twitter posts to give a quick overview of main topics or locations (52) (*n* = 3). Several papers describe the use of distinct sub-sections of the page like sidebars (37) or tabs (53) (*n* = 9) which facilitate the navigation.

Nineteen papers describe the use of color to further enhance understanding. Some of them explicitly use the traffic light colors—green, amber and red—to take advantage of the popular associations regarding these colors (54). In some areas—as described by Bernard et al. (55) for the medical sector—it is beneficial to use color codes that are prominent in a certain work environment (e.g., black for “death of disease”) (see Appendix E).

#### 3.1.5. Functions that dashboards offer besides the pure visualization of information (RQ 1.5)

Dashboards are not only used for the visualization of data but offer further functions, features and components depending on the situation or task at hand. These include, for example, the possibility to look at data representing longer time scales (56) (*n* = 13) or to conduct predictive analysis (57) (*n* = 4). The possibility of data customization is described in almost half of the papers considered in the review. This includes the possibility of (a) selecting and filtering datasets (58) (*n* = 27), (b) searching for datasets of variables (38) (*n* = 8), and (c) sorting or grouping data (59) (*n* = 4).

In addition, ten papers describe dashboards that offer direct export e.g., of data files, screenshots (50) or reports (60). These downloads can be used for in-depth analysis, as illustrative material in meetings, or they can be uploaded into other tools for further use (61). For participatory dashboards that rely on data from sources such as the public or medical staff (62), the possibility to directly add data to the dashboard is an important function. Data entry is provided through web-based report files (63), customized online forms, via posts or SMS and some dashboards provide direct data upload (64). To further enhance user experience, data can be copied and edited (65) (*n* = 22). Eight papers note that an alarm function is particularly useful for dashboards on crisis management, which allows users to receive messages about alarming situations or noteworthy developments via SMS or email (66). Seven dashboards make use of apps to display alerts or to report data (64).

To facilitate cooperation and communication between dashboard users, dashboards can offer the possibility to communicate within the dashboard (67) via discussion forums, messaging and comments (68) (*n* = 10).

Over one third of the described dashboards offer possibility to customize the visualization of the dashboard (*n* = 24). Especially zooming in or out of maps and drilling down to a specific region, for example, enables the user to explore the data in detail (47) (*n* = 12). Moreover, modifying templates, charts and other visual elements enhances user experience (59) (*n* = 3) (see Appendix F).

### 3.2. Using dashboards in public health: challenges and objectives (RQ 2)

In terms of RQ 2, dashboard objectives offered answers to public health challenges. First, we will sketch these public health objectives and challenges. Second, the objectives and challenges of public health dashboards described in the study sample will be outlined (see Appendix G).

#### 3.2.1. Public health challenges addressed (RQ 2.1a)

##### 3.2.1.1. Challenge: data collection for developing and implementing interventions

The first challenge addresses the identification of health threats by adequate surveillance/monitoring systems. These health threats can be classified into three categories. In some of the articles examined, the disease is explicitly associated with certain risks or vice versa, leading to counting in several categories (see Appendix H):

a) Risks such as obesity (69), environmental pollution (70), food contamination (64), or injuries (51) (*n* = 18);
b) Communicable/infectious diseases like Dengue Fever (33) or reproductive tract infections (62) (*n* = 29) as well as non-communicable diseases like cancer (55) or dementia (71) (*n* = 10);
c) Emergencies such as natural catastrophes (72) or human-made disasters (73) (*n* = 17).

All three kinds of health threats are a global issue beyond political borders due to rising cross-border mobility, poverty or climate change. This requires an alignment of data: So far, missing or not transferable data makes it difficult to identify new diseases (58), to track and explore these diseases (74) as well as to develop strategies eliminating causes for illnesses or death (54).

##### 3.2.1.2. Challenge: communication management and the use of information and communication technology

Related to the first challenge is the question of how to manage the vast amount of produced and collected information in public health. All articles included in the sample deal in one way or another with time, effort and cost as a key challenge in dealing with the high volume of data and its digitisation. Articles critically addressed an insufficient use of health-related ICT solutions in (1) monitoring social disparities leading to higher mortality and morbidity rates (62), (2) enabling access to health care as a marginalized community (64), or (3) dealing adequately with mis- and disinformation (52, 75).

Furthermore, a lack of training for health workers was stated—leading to an improper use of digital tools (75). These shortages result in (a) a poor management of scarce resources (72), (b) missing target group specific evidence-based communication strategies including the tracking of health issues as an objective (52) and (c) inefficient decision-making (76) leading to high economic and social costs.

#### 3.2.2. Public health objectives pursued (RQ 2.1b)

Four main public health objectives could be identified to tackle these challenges. (A) Threatening situations shall be controlled, for example, through surveillance or risk prevention (77) (*n* = 40). (B) Information management has to be improved (*n* = 26) by for instance enhancing knowledge (75) or addressing target groups (78). C) Quality of life has to be enhanced (*n* = 17) by improving health care and services, e.g., through health promotion (39) or risk reduction (79). D) And in response to threatening situations, public health policies resp. measures have to be adjusted (*n* = 16): Policy programs focusing on health promotion, for example, need to be sustainable and long-term, community protection initiatives need to be supported, and digital tools for efficient decision-making need to be implemented as well as their access guaranteed (42).

#### 3.2.3. Specific technological or administrative challenges related to the use of public health dashboards (RQ 2.2a)

Besides the distinctive objectives of public health dashboards, the reviewed literature also helps to extract various challenges (see Appendix G) that might be of relevance while constructing, using or deploying dashboards in a public health context. The identified dashboard challenges refer to (a) the visualization and processing of the data (*n* = 46), (b) the development of the dashboard (*n* = 7) and (c) the use of the dashboard (*n* = 9).

##### 3.2.3.1. Challenges regarding the visualization and processing of data

First and foremost, the identified literature focused on different challenges associated with the visualization as well as the complexity, integration, quality and analysis of data. Zhu et al. (53), for example, underline the challenge that data visualizations need to be adaptable to different usage patterns as well as scenarios, while Zheng et al. (80) accentuate the need of accurate, visual information summarization for an appropriate understanding of e.g., crises or outbreak events. This last aspect already points to another challenge, associated with the development and use of public health dashboards: the complexity of visualized data. Husain et al. (59) note that the complexity and heterogeneity of (big) data may ultimately constrain the use of established methods, tools and services. In this context, challenges regarding the construction of dashboards may especially involve the need to tackle possible information overload (76), associated with e.g., data redundancy or the amount of information, received by a respective dashboard system (44). Corresponding with this finding, another issue described in the reviewed literature is the integration and transfer of data from diverse and heterogeneous sources. Data collected through different systems such as spreadsheets, via email or non-interoperable systems could cause serious problems in regards to its integration in a coherent dashboard system (65). Lack of standards or unstructured data formats, often coming from different sources (76), may ultimately inhibit holistic data understanding and interpretation (59). In addition, the reviewed articles highlighted that there are challenges in designing dashboards in terms of data quality, especially in the context of public health. In this context, Vila et al. (40) note diverse challenges such as data accuracy (66) and consistency as well as ensuring and fulfilling the legally required regulations on data protection. Lastly, the literature also frequently discussed challenges regarding the analysis of data. Rees et al. (37) accentuate that the type of surveillance method employed by involved response units (for example in infectious diseases control) can lead to an under- or overestimation in observed prevalence. Recently, and especially concerning dashboards that integrate data from diverse social media platforms, misinformation has been noted as a major problem, compromising data analysis (52). In line with this, the time needed to analyze visualized data may also pose a major challenge in dashboard design (68).

##### 3.2.3.2. Challenges regarding the development of the system or dashboard

Further challenges discussed in the reviewed literature were concerned with the development of the system incorporating a dashboard or the dashboard itself. A concern that was selectively addressed in the identified literature has been the cost effectiveness in regards to a specific dashboard and its system architecture (81). Moreover, the use and design of dashboards in a public health context also faces legal challenges in particular, as pointed out by Vila et al. (40). As already mentioned, the design of dashboards and the use and visualization of specific data needs to be aligned with and fulfill respective government regulations and laws.

##### 3.2.3.3. Challenges regarding the use of dashboards

Other major challenges that have occasionally been discussed in the reviewed articles relate to the actual use of a dashboard. In this context, the articles particularly highlight challenges with regard to the use of a corresponding dashboard by specific user groups. Key aspects in this context were that the dashboard itself is “user-friendly” (44), implying the need to design dashboards that are easy to understand, appealing and intuitive. Appropriately designed systems should take the information-seeking behavior of respective user groups and their respective health literacy skills into account (82), as these aspects may ultimately affect the utilization of a dashboard and the interpretation of its visualized and aggregated data sets. Furthermore, and with special regard to participatory dashboards, the design of a dashboard system needs to be concerned with securing the pro-active participation of focal user groups (68).

#### 3.2.4. Specific technological or administrative goals related to the use of public health dashboards (RQ 2.2b)

Besides underlining the challenges associated with the development or use of public health dashboards, the reviewed literature also helps to identify objectives that are specifically linked to the use of dashboards in a public health context (see Appendix G). Overall, the objectives that are discussed in the literature can be grouped into four main categories, underlining the aims that are hoped to be achieved by implementing or using a dashboard: (a) improving surveillance and monitoring (*n* = 49), (b) improving (crises) management procedures as well as inter-agency coordination (*n* = 22), (c) providing (public) access to information (*n* = 18) and, finally, (d) enabling participation (*n* = 8).

##### 3.2.4.1. Improve surveillance and monitoring of public health risks or crises

The literature reviewed primarily highlights the function of dashboards to improve the monitoring and surveillance of, for example, infectious disease outbreaks. Benson et al. (83) note that dashboards might support involved response units in situational awareness and collaborative decision-making. In this context, the cross-verification (68) and early warning (34) of outbreak or other adverse events as well as the possibility to trace back and rapidly detect respective crises situations (74), were repeatedly underlined as objectives of data visualization as well as aggregation via dashboards. However, the discussed dashboards are not just limited to the immediate surveillance of crises events, but also aim at the prediction of outbreaks and other adverse events, as was noted for the dashboard, focused on in Jamil et al. (77). More so, dashboards aim to present relevant information and thus reduce time spent searching for information (44).

##### 3.2.4.2. Improve (crises) management procedures and inter-agency coordination

The above mentioned factors associated with the improvement of surveillance and monitoring ultimately correspond to another, frequently discussed objective of public health dashboards: the improvement of (crises) management procedures. In this context, public health dashboards support decision-making under high time pressure and thus reduce the time needed for effectively mitigating the effects of outbreak events (63). In addition, they improve inter-agency coordination or cross border surveillance (58) by combining and aggregating data from agencies with different mandates (37). Furthermore, dashboards may as well facilitate information sharing between different actors.

##### 3.2.4.3. Provide (public) access to information

The legitimation of political-administrative decision-making by means of data visualization through public health dashboards played a marginal role in the reviewed literature and, even more so, was not mentioned as a particular objective of information provision. Nevertheless, the relevance of public access to certain information was discussed in a fraction of evaluated articles—both for non-professionals and citizens as well for special user groups, such as public health experts and professionals (64). Associated with this, Thomas and Narayan (62), for example, discussed the relevance of dashboards for supporting the health of citizens by increasing access to health related information and allowing to understand crises situations across space and time (37).

##### 3.2.4.4. Enable participation

In addition to the mere access to or the reception of relevant information, reviewed articles have occasionally also noted the active involvement and inclusion of user groups in order to support the surveillance and management of infectious disease outbreaks or public health in general. Tegtmeyer et al. (74), for example, cite the general participation of users as a distinctive objective of their focal dashboard. Moreover, Rees et al. (37) explicitly note the involvement of users in reporting—in this case: of suspect animals—as an objective of their dashboard.

### 3.3. Information needs when engaging with public health dashboards (RQ 3)

The findings presented in the following are based exclusively on the assessment of the eighteen identified user studies. We refrain here from quantifying aspects and thus from stating item numbers in relation to the various information needs. This particular caution is mainly due to the fact that relevant terms such as “ease-of-use” or “usability” were often not operationalised consistently or at all in the evaluated articles. This in turn has made it difficult to compare the results of the different articles in a meaningful way. At the same time, however, specific article numbers are not given here, as a small *n* could imply that a certain aspect was not as relevant as others were, although this often does not have to correspond to its actual relevance, but can also be related to the focus of the studies and the overemphasis on other aspects.

Although the information needs of specific user groups may vary due to the diversity of dashboards (see Appendix I), a number of studies have identified similar core criteria.

The ease of use was one aspect frequently mentioned in the studies. The user must be able to use the dashboard intuitively. Some applications require technical understanding or a certain literacy as well as skills and qualifications of the users, which influences their acceptance of the dashboard and its implementation into the workflow (47). As described by Hamoy et al. (75), it is beneficial to train the staff or users of the dashboard, e.g., through workshops. The provision of a hotline can be another way to improve acceptance and ease of use (75). Furthermore, the technical devices should allow for easy handling of the application. Usability is otherwise limited (e.g., small screen for displaying complex tables).

Besides the qualifications of the users, the compatibility of the dashboard with the work environment of the user is crucial for its successful implementation. Several papers describe the demand that dashboards have to be compatible with the users' workflow. This implies that its use (a) does not entail more work but facilitates specific work steps like data collection, updates or analysis while also (b) saving time (42, 75). The latter often includes the need to work with real-time data. Thus, saving time refers to both, finishing a task in less time but also saving time in the provision of data. The application should allow the quick update of data (61). There are also additional delays when data needs to be validated or verified. Dashboards that can be accessed independent of time and place are particularly convenient (84).

Rural areas are a particular challenge with regard to the collection of data, as the infrastructure is not always in place and developers have to plan with fewer employees, lack of electricity, poor internet reception, and inadequate availability of technology (75). In this case, the question of how users can access and enter the data is a particular challenge.

Several aspects can enhance the engagement with a dashboard and facilitate the usage. For example, several papers state that users wish for interactive features such as notifications. These can be used to inform the user about news on the dashboard or can pop-up whenever a task, such as a data upload, is completed. Besides notifications, the possibility of networking is mentioned to be a helpful and often requested feature of a dashboard (61, 85). Depending on the requirements, networking can include a messaging tool, the possibility to share data or a way to comment on or reply to other users' posts or other forms of input (85).

As described above, a multitude of visual elements is used in the dashboards. However, the use of different elements and colors is rarely evaluated in detail. More often, studies describe the overall success of the dashboard. It can be noted that the use of colors seems to facilitate understanding and is mostly intuitively understood [e.g., red for danger or severity, see Bernard et al. (55)].

### 3.4. Mixed Methods Appraisal Tool

Of the eighteen studies that were explicitly considered as user studies (and thus considered in the critical appraisal stage via MMAT), eight articles exclusively applied qualitative methods, while seven articles were decidedly quantitatively oriented in their study approach (see Appendix J). Three articles employed a mixed methods approach by combination of qualitative and quantitative methods. Our sample included neither randomized controlled trials nor non-randomized studies. Surveys were the method most often used in the quantitative studies. However, the insufficient description of the sample and target groups in some articles sometimes did not allow for an accurate assessment of the representativeness of the survey sample for the target population.

Moreover, in most cases, a final assessment concerning the risk of nonresponse bias as well as the appropriateness of the studies' statistical approaches was confounded by the lack of necessary data or information in the respective papers. In regards to the qualitative studies, interviews, were the most frequently used method. However, in some cases, authors simply stated, that they had received “input” from an unspecified group, which made it difficult to clearly evaluate the methods being used in these studies. Other methods used were focus groups as well as participant observations.

All in all, the quality appraisal of included studies by means of the MMAT yielded an average overall rating score of 40%, indicating a rather moderate average methodological quality of the eighteen studies considered in the quality appraisal step of our literature review.

However, significant differences in overall quality can be observed between the different types of studies. With regard to the qualitative oriented studies considered in this step of our literature review, a quality range of 20 to 100% can be noticed, whereby the average score for qualitative studies was 55%, suggesting a score higher than the overall average score. Assessing the quantitative studies as well as studies with a mixed-methods design, we see a considerably lower mean value with regard to the respective study quality (qualitative studies: 29%; mixed-methods studies: 27%). However, these final assessments should be approached with caution, since we had to select “Can't tell” at least once in each study, except for two qualitative oriented studies. As was discussed above, this indicates that critical or relevant data, required for a final assessment on a certain item, is often missing. This deficit, however, points to a general problem of methodological reporting in empirical studies, which is why a comprehensive and accurate appraisal of included studies is often more difficult than anticipated.

## 4. Discussion

Assuring public health in a world that is confronted with ever changing challenges due to globalization, climate change and various other developments demands for adapted technologies. The results of this literature review show that dashboards cover a wide range of public health issues—from foodborne diseases to environmental hazards (see Appendix B), and provide data for different target groups such as medical experts, researchers, or specifically concerned communities. Dashboards have become an important tool for communicating health risks through the visualization of data—offering options such as (near) real-time monitoring or retrieving data from a variety of sources ranging from health authorities on different levels, healthcare organizations to research organizations and the media. The dashboards addressed public health objectives in at least one of the four dimensions: Controlling threatening situations, improving information management, enhancing quality of life and adjusting public health policies and measures (see Appendix H).

This review examined 65 papers that allowed conclusions to be drawn about the objectives and challenges of public health communication via dashboards. In total 18 of them also provided user research and information on the user needs. Most of the papers emphasized that dashboards enable users to add, enter, copy or merge data followed by data export opportunities and data analysis. Involving users and enabling their (continuous) participation thus not only forms an objective of information provision via dashboards themselves, but also aims at supporting and improving the surveillance and management procedures, thereby improving public health surveillance. Linked to this is the argument that detection, prediction and the management of outbreaks will become easier. Dashboards provide a timely and accurate overview of the situation and automatically notify the user of alerts. We can conclude that the overall aim is thus to raise the situational awareness of health professionals, politicians and citizens in general.

Secondly, communication (management) processes can be improved through data reporting and sharing as well as specific data visualizations such as maps or graphs. Here our systematic review sheds light on the specific challenges faced by dashboard developers. These range from the integration and transmission of data from different and heterogeneous sources, to the alignment of data with legal requirements, data accuracy, as well as appropriate and comparable surveillance methods (see Appendix G). Interestingly, dashboards that work with social media data are particularly challenged when it comes to misinformation. As for the role of misinformation in crises (51), this is a research gap that definitely needs to be addressed.

Design is a challenge and essential: Maps showing disease or risk distribution and diagrams in all their variations play the most important role—often combined with questions of color use. Graphics, animations, or audio-visual means such as social media streams or videos were less frequently reported. Although a variety of visual elements are used in the dashboards, a detailed evaluation of these elements is missing, especially an evaluation of the interdependencies of different modes such as layouts or color. This is consistent with research gaps identified by Berg et al. (16). The compositionality of these individual modes can produce a different meaning compared to analyzing the modes separately (86). In addition, and given that somewhat more than a third of the articles included in the review describe how users can customize the visualization of the data, a related research question for future studies would have to be: How do dashboard users interpret the visualized data and make an overall coherence between the interacting modes? This also refers to the long-held recognition that users, as recipients, need to be seen as active participants who contribute content (87), draw their conclusions from the data on risks and take protective measures if necessary, or may misjudge risks, for example due to a lack of health literacy.

Another finding of this review also concerns the role of users in improving access to information through dashboards. Those studies considering the specific challenges and objectives from a technological, administrative, as well as a user perspective made evident how dashboards increase access to health related information and enable an understanding of critical public health issues (37, 62). Important for understanding the data, however, is health literacy, which is very rarely addressed in the sample studied. This also corresponds to existing research gaps identified so far and demands for future socio-technical research (13, 88).

One aim of this literature review was to identify information needs of dashboard users (see Appendix I). However, most studies are limited to describing the process of technical construction and design of a particular dashboard (*n* = 47). A comparatively small number of publications deal explicitly with the reception of dashboards by users (*n* = 18). Furthermore, some of these studies are limited to a purely functional evaluation of the dashboard by the respective development teams without applying user-centered design approaches. Identifying information needs by using risk communication models such as the Health Belief Model or the Extended Risk Assessment Model is the exception (58, 62). Relevant constructs such as risk perception, perceived severity and self-efficacy as well as existing concepts such as health literacy, numerical literacy and data visualization literacy (88) are not sufficiently taken into account to provide insights for data visualization and thus increase the comprehensibility of the data. Thus, the sample did not provide sufficient information on whether the dashboards meet the requirements of the respective users. This is consistent with the findings of reviews looking at public health dashboards (11, 89) revealing a relevant research gap, which should be taken into account for future projects. Accordingly, it can be concluded that a user-driven development strategy, theory- and evidence-informed, is key to developing a user-friendly design by capturing key information through a user-friendly interface design, for example by collecting data on perceived ease of use and perceived usefulness.

Precisely because public and scientific institutions also want to reach the public via an open data policy with the dashboard they created in connection with the COVID-19 pandemic (35), these gaps need to be explored. One way to do this is to use known communication models on information behavior to survey information needs and to take the corresponding results into account when designing the user interface.

### 4.1. Limitations

One limitation of the analysis of the papers was the inconsistent differentiation of the term “dashboard”. While some papers only refer to dashboards as the visual representation of data (63), others describe entire systems that include various functions, as dashboards (73). We applied the understanding of the term that was expressed in the respective papers to our analysis.

As already described, the papers report little on their methodological approach. Accordingly, the educational effect for other researchers is limited. Even more than a shortcoming of the respective authors, we see a possible reason in the restrictive publication requirements of some journals, which make a detailed description of the methods difficult or even impossible.

Although a systematic approach in retrieving articles on public health dashboards was followed, we cannot guarantee that all eligible studies offering answers to the research questions were found. Firstly, we limited the number of years (2010–2020) and databases. Since we limited the field to dashboard solutions that are scientifically covered, the overview (Appendix B) does not provide information on all existing public health dashboards. Secondly, we had to differentiate between a user study and a descriptive one including brief communications articles as well as developer studies—excluding studies that only focus on predictive models instead of developing a real dashboard. There may be studies in which the difference between modeling and developing is very small. Thirdly, we conducted a review that explicitly aimed at papers from various scientific disciplines. The article followed specific rules of writing and structuring articles resulting in challenges to compare data, reporting, etc. Finally, we reviewed data reported in included studies. We did not request any further data by contacting the first authors.

## 5. Conclusion: implications for dashboard research

The aim of our systematic review was firstly to identify the public health challenges and objectives that were displayed by dashboards between 2010 and 2020. Analyzing the visualization of data and included functions, we aimed to outline solutions that dashboards offer as a specific digital health technology. Secondly, the review aimed to evaluate the empirical studies that focused on the needs of the users by applying the MMAT. Although dashboards have come to play an important role in data-based visualization of public health issues, particularly due to their use during the COVID-19 pandemic, the number of publications explicitly addressing user reception of dashboards is small. As a specific form of data visualization, dashboards are of particular importance—especially, when detecting and monitoring risks and crises and their effects on public health.

The dashboards studied reflect the challenges identified in the field of public health in relation to technological progress. They enable faster data collection, sharing and analysis of data. However, one identified research gap seems to be very important with regard to the usefulness of this risk and crisis communication tool. If the needs of users in the context of health information behavior are not sufficiently empirically investigated, the benefits of dashboards for risk reduction or risk behavior change will remain without evidence. This point goes hand in hand with the need to examine the information behavior of specific target groups based on existing and valid theoretical models and to think about multimodality in meaning-making.

Applied research would benefit (a) from including risk communication models and constructs such as scientific literacy as well as different disciplinary perspectives (e.g., IT, communication studies, psychology) and (b) from a more inclusive approach that involves potential target users throughout the construction and design process. For this, a pre-design consideration of risk information needs that potential target groups might have is essential.

## Data availability statement

The original contributions presented in the study are included in the article/Supplementary material, further inquiries can be directed to the corresponding author/s.

## Author contributions

AS: conceptualization (main idea and theory), project administration, methodology (design and operationalization), data collection, data analysis, writing—original draft, and writing—review and editing. FB: methodology (design and operationalization), data collection, data analysis, writing—original draft, and writing—review and editing. JG: data analysis, writing—original draft, and writing—review and editing. G-FB: conceptualization (main idea and theory) and writing—review and editing. All authors contributed to the article and approved the submitted version.
