# Supplementary material for: Digital dashboards visualizing public health data: a systematic review
Source: Front Public Health. 2023 May 4;11:999958. doi: 10.3389/fpubh.2023.999958 (PMC10192578; doi:10.3389/fpubh.2023.999958)
Supplement: Supplementary file 1 [file Data_Sheet_1.PDF]

Inclusion and Exclusion Criteria and Their Rationale

Criteria	Rationale/Comments/Thoughts
**Inclusion Criteria**	
I1 written in English	
I2.1 *search string 1:* peer-reviewed journal articles, conference proceedings/papers, anthology contributions/serials	- conference proceedings in computer science, design and engineering have become the primary way to publish research results in these disciplines; regarding our topic, it is necessary to include these disciplines to get more information on the state of art in digital health communication of crises etc.<br>- to proof by sample
I2.2 *search string 2:* peer-reviewed journal articles, conference proceedings/papers, anthology contributions/serials + brief communications, correspondences	- the second search string further accounts for brief communications and correspondences (e.g. letters), as substantial publications (e.g. journal articles, conference proceedings, etc.) on SARS-CoV-2-related dashboards are still scarce
I3 publications have to be published between 2010-2020	- only studies published from 2010 onwards will be included in order to reflect technological developments associated with the above-outlined review questions<br>- displaying complex data sets by resorting to interactive modes of visualization (e.g.) has especially been enabled by Web 2.0-technologies, which is why the review only considers relevant research findings from the last ten years
I4 Publications have to contain the elements of the main research question that are either the (diverging) information needs for public health dashboards regarding risk understanding or general design and functional aspects of public health dashboards	- if there is a link between animal and human health risks like in the case of a zoonotic disease, this will also be included

I5 publications have to display or visualize public health data linked to human diseases, pandemics, outbreaks, crises, risks, urban, occupational and environmental health in a (more or less) cohesive and specific dashboard: from risk prevention, early detection, crisis management to aftermath	- if a clinical dashboard also includes public health data, e.g. of the region, the article shall be included<br>- non-communicable diseases (e.g. diabetes) and communicable diseases (e.g. Ebola) and their effects on or consequences for public health<br>- climate change as well as natural disasters and their consequences for public health, e.g. flooding<br>- besides the environmental determinants of disease, social determinants of diseases, e.g. effects of workplace conditions, are also linked to public health<br>- different types of data: epidemiological data as well as social media data<br>- visualizations of data are described on the basis of a specific dashboard
**Exclusion Criteria**	
E1 answer is no to either I1, I2, I3, I4, I5	
E2 published literature reviews	
E3 papers that focus medical visualization techniques in an explicit medical context	- if the focus is especially on medical imaging and medical visualization techniques like mammography, it will not support finding answers to the research question complex asking for results in visualizing health risks rather than diagnoses
E4 papers to which we had no full access	- author contacted; not answering within four weeks
E5 papers dealing with dashboards focusing on disaster coordination, explicitly regarding casualty management systems	- e.g. “communication between medical incident commanders (ICs) and first medical responders (paramedics) at mass casualty incidents (MCI) sites”
E6 papers focusing on monitoring individual health data (or the visualization of personal health data only used by patients, their respective physicians or health providers)	- if an article about a personal health dashboard that refers to a public health disease does not focus on the effects on or consequences for public health, it will be excluded

	- if, for instance, a physician works with a dashboard that visualizes individual health data (e.g. a diabetes patient) and the dashboard offers an option to compare the individual data with public health data regarding the disease and if the physician only uses the compared data to inform the patient about possible risks, then the article will be excluded (dimension of public health missing)<br>- if the article focuses on health and well-being technologies, the article will be excluded
E7 papers focusing on performance indicators	- e.g. unit-specific nursing performance; comparison between several nation-states regarding health system performance indicators<br>- e.g. acute care coordination that focuses on the individual and fast treatment of patients in hospitals
E8 papers focusing on clinical decision management systems or acute care coordination	- e.g. papers focusing on physicians'/nurses' information needs, on clinical throughput or the review process by physicians regarding the effectiveness of patient treatment
E9 papers exclusively focusing on technical aspects	- e.g. if weather sensors are tested that provide data to a dashboard system
E10 papers focusing on serious games and gameplay data	
E11 exclusively theoretical papers	- e.g. papers which make no reference to a specific dashboard or papers which are not application-oriented
E12 papers not elaborating on dashboards	- just mentioning or referencing data from a dashboard
E13 papers dealing with dashboards that only focus on the implementation of (health) policy guidelines	
