# Supplementary material for: Digital dashboards visualizing public health data: a systematic review
Source: Front Public Health. 2023 May 4;11:999958. doi: 10.3389/fpubh.2023.999958 (PMC10192578; doi:10.3389/fpubh.2023.999958)
Supplement: Supplementary file 2 [file Data_Sheet_2.pdf]

Appendix B

Author (Publication Year)	Dashboard: Name / Denotation	Study Type	Country of Author	Dashboard Focus	Geographical Monitoring
**Al Manir, M. S., et al. (2018). A Surveillance Infrastructure for Malaria Analytics: Provisioning Data Access and Preservation of Interoperability. *JMIR Public Health And Surveillance*, *4*(2), Article e10218.**	Semantics, Interoperability, and Evolution for Malaria Analytics (SIEMA) platform	Descriptive	United States of America and Canada	Infectious Diseases I Malaria	International
**Alvarez, V. C., et al. (2019). Visualization of Health Data. In J. C. W. Lin, I. H. Ting, K. Wang, & T. Tang (Eds.), *Multidisciplinary Social Networks Research, 6th International Conference, MISNC 2019, Wenzhou, China, August 26–28, 2019, Revised Selected Papers* (Vol. 1131 CCIS, pp. 118-130): Springer.**	Dashboard on environmental factors and there connection with public health	Descriptive	United States of America	Diseases I Pollution I Environment	International
**Avvenuti, M., et al. (2018). CrisMap: A Big Data Crisis Mapping System Based on Damage Detection and Geoparsing. *Information Systems Frontiers*, *20*(5), 993–1011.**	CrisMap	Descriptive	Italy	Crisis I Disaster	National
**Basdere, M., et al. (2019). Safe: A Comprehensive Data Visualization System. *INFORMS Journal on Applied Analytics*, *49*(4), 249-261.**	SAFE	Descriptive	United States of America	Crisis I Emergencies I Mass Events	Local
**Benson, A. L., et al. (2010). *Adaptive Development of a Common Operating Environment for Crisis Response and Management.* ISCRAM 2010 – 7th International Conference on Information Systems for Crisis Response and Management: Defining Crisis Management 3.0, Proceedings, Seattle, WA: Information Systems for Crisis Response and Management, ISCRAM.**	Puget Sound Common Operating Environment (PSICOE)	Descriptive	United States of America	Crisis I Emergencies	Local
**Bernard, J., et al. (2019). Using Dashboard Networks to Visualize Multiple Patient Histories: A Design Study on Post-Operative Prostate Cancer. *IEEE Transactions on Visualization and Computer Graphics*, *25*(3), 1615-1628.**	Dashboard Networks on Patient Histories – Prostate Cancer	User Study	Germany and United Kingdom	Diseases I Cancer	International
**Bhardwaj, S., et al. (2014). Elimination of Mother-to-Child Transmission of HIV in South Africa: Rapid Scale-up Using Quality Improvement. *South African Medical Journal*, *104*(3), 239-243.**	Dashboard to monitor mother-to-child-transmission of HIV	Descriptive	South Africa	Infectious Diseases I HIV	National
**Braa, J., et al. (2017). Health Information Systems in Indonesia: Understanding and Addressing Complexity. In M. S. Islam, F. Wahid, J. E. Priyatma, J. Choudrie, & J. M. Bass (Eds.), (Vol. 504, pp. 59-70): Springer New York LLC.**	Dashboard to coordinate Health Information Systems	Descriptive	Norway	Health Related Services	National

**Brownson, R. C., et al. (2015). Applying A Mixed-Methods Evaluation to Healthy Kids, Healthy Communities. *Journal of Public Health Management and Practice*, *21*, 16-26.**	HKHC Community Dashboard	Descriptive	United States of America	Diseases I Obesity<br>Health Related Services	International
**Campbell, T. C., et al. (2014). Development of the Respiratory Disease Dashboard for the Identification of New and Emerging Respiratory Pathogens. *Johns Hopkins APL Technical Digest (Applied Physics Laboratory)*, *32*(4), 726-734.**	Respiratory Disease Dashboard	Descriptive	United States of America	Infectious Diseases	International
**Carmichael, J. M., et al. (2017). Leveraging Electronic Medical Record Data for Population Health Management in the Veterans Health Administration: Successes and Lessons Learned. *American Journal of Health-System Pharmacy*, *74*(18), 1447-1459.**	VISN 21 Clinical Dashboards	Descriptive	United States of America	Health Related Services I<br>Veterans' Health	National
**Choudhary, V., et al. (2020). *AirQ: A Smart IOT Platform for Air Quality Monitoring.* 2020 IEEE 17th Annual Consumer Communications & Networking Conference (CCNC), Las Vegas, NV.**	AirQ	Descriptive	Singapore	Diseases I Pollution I Airborne	Local
**Concannon, D., et al. (2019). Developing a Data Dashboard Framework for Population Health Surveillance: Widening Access to Clinical Trial Findings. *JMIR Formative Research*, *3*(2), Article e11342.**	Data Dashboard Framework for Population Health Surveillance	User Study	United Kingdom and South Africa	Health Related Services I<br>Health Promotion Programs I<br>Public Health Interventions	National
**Devi, L. N., et al. (2018). *Live Demonstration on Smart Water Quality Monitoring System Using Wireless Sensor Networks.* 2018 IEEE SENSORS, New Delhi, India.**	Smart Water Quality Monitoring System	Descriptive	India	Diseases I Pollution I Waterborne	Local
**Dong, E., et al. (2020). An Interactive Web-Based Dashboard to Track COVID-19 in Real Time. *The Lancet. Infectious Diseases*, *20*(5), 533-534.**	COVID-19 Dashboard	Descriptive	United States of America	Infectious Diseases I Corona	International
**Erraguntla, M., et al. (2012). *Open Source Text Based Biovigilance.* Proceedings of the 2012 International Conference on Artificial Intelligence (ICAI 2012, Vol. 1), Las Vegas, NV.**	Threat Assessment Dashboard (BioTHAD)	Descriptive	United States of America	Infectious Diseases	National
**Estuar, M. R. E., et al. (2016). *The Challenge of Continuous User Participation in eBayanihan: Digitizing Humanitarian Action in a Nationwide Web Mobile Participatory Disaster Management System.* 2016 3rd International Conference on Information and Communication Technologies for Disaster Management (ICT-DM), Vienna, Austria.**	eBAYANIHAN	User Study	Philippines	Crisis I Disaster	National
**Federico, L., et al. (2016). *SINSE+: A Software for the Acquisition and Analysis of Open Data in Health and Social Area* 24th Italian Symposium on Advanced Database Systems (SEBD 2016), Ugento, Lecce, Italy.**	SINSE+	Descriptive	Italy	Health Related Services I<br>Citizen Wellness	Local
**Gourevitch, M. N., et al. (2019). City-Level Measures of Health, Health Determinants, and Equity to Foster Population Health Improvement: The City Health Dashboard. *American Journal of Public Health*, *109*(4),**	City Health Dashboard	User Study	United States of America	Health Related Services I<br>Urban Health	Local

**585-592.**					
**Hamoy, G. L., et al. (2016). Real-Time Regular Routine Reporting for Health (R4health): Lessons from the Implementation of a Large Scale Mobile Health System for Routine Health Services in the Philippines. *Acta Medica Philippina*, *50*(4), 280-294.**	R4Health	User Study	Philippines	Health Related Services I Health Reporting System	Local
**Harris, J. K., et al. (2018). Evaluating the Implementation of a Twitter-Based Foodborne Illness Reporting Tool in the City of St. Louis Department of Health. *International Journal of Environmental Research and Public Health*, *15*(5), Article 833.**	HealthMap Foodborne Dashboard	User Study	United States of America	Diseases I Foodborne	Local
**Hoare, G., et al. (2010). Developing H1N1 Hospital Surge "Dashboard" Indicators: A Demonstration. ISCRAM 2010 - 7th International Conference on Information Systems for Crisis Response and Management: Defining Crisis Management 3.0.**	H1N1 Hospital Surge Dashboard	Descriptive	United States of America	Infectious Diseases I H1N1-Pandemic	Regional
**Homsuwan, P., et al. (2018). Visualization Development of Health Data Reporting with Business Intelligence Techniques. *Journal of the Medical Association of Thailand*, *101*(6), 49-54.**	Business intelligence dashboards for health data	User Study	Thailand	Health Related Services	National
**Husain, S. S., et al. (2015). SOCR Data Dashboard: An Integrated Big Data Archive Mashing Medicare, Labor, Census and Econometric Information. *Journal of Big Data*, *2*(1), Article 13.**	SOCR Data Dashboard	Descriptive	United States of America	Health Related Services	National
**Husain, W., et al. (2016). M-DENGUE: Utilizing Crowdsourcing and Teleconsultation for Location-Based Dengue Monitoring and Reporting System. *Jurnal Teknologi*, *78*(9-3), 89-95.**	M-DENGUE	Descriptive	Malaysia	Infectious Diseases I Dengue	National
**Jamil, J. M., et al. (2016). An Innovative Data Mining and Dashboard System for Monitoring of Malaysian Dengue Trends. *Journal of Telecommunication, Electronic and Computer Engineering*, *8*(10), 9-12.**	Dengue Fever and Dengue Hemorrhagic Fevers Monitoring System	Descriptive	Malaysia	Infectious Diseases I Dengue	National
**Jinpon, P., et al. (2017). Integrated Information Visualization to Support Decision Making for Health Promotion in Chonburi, Thailand. *Walailak Journal of Science and Technology*, *16*(8), 551-560.**	Dashboard Decision Support System	User Study	Thailand	Health Related Services I Health Promotion Programs	Local
**Jinpon, P., et al. (2017). Integrated Information Visualization to Support Decision-Making in Order to Strengthen Communities: Design and Usability Evaluation. *Informatics for Health & Social Care*, *42*(4), 335-348.**	Community Well-Being Assessment System	User Study	Thailand	Health Related Services I Health Promotion Programs	Local
**Kamadjeu, R., et al. (2017). Designing and Implementing an Electronic Dashboard for Disease Outbreaks Response - Case Study of the 2013-2014 Somalia Polio Outbreak Response Dashboard. *The Pan African medical journal*, *27*.**	Somalia Polio Outbreak Response Dashboard	Descriptive	Kenya	Infectious Diseases I Polio	National

**Kostkova, P. (2013).** ***A Roadmap to Integrated Digital Public Health Surveillance: The Vision and the Challenges.*** **WWW '13 Companion Proceedings of the 22nd International Conference on World Wide Web, Rio de Janeiro, Brazil.**	Digital Public Health Dashboard	Descriptive	United Kingdom	Infectious Diseases I Communicable Disease Outbreaks I SARS	International
**Kostkova, P., et al. (2014).** ***Integration and Visualization Public Health Dashboard: The Medi+Board Pilot Project.*** **WWW '14 Companion: Proceedings of the 23rd International Conference on World Wide Web, Seoul, Korea.**	medi+board	Descriptive	United Kingdom	Infectious Diseases I Communicable Disease Outbreaks I Swine Flu	International
**Lee, M. T., et al. (2020). Web-Based Dashboard for the Interactive Visualization and Analysis of National Risk-Standardized Mortality Rates of Sepsis in the US.** ***Journal of Medical Systems*****,** ***44*****(2), Article 54.**	Web-based Dashboard for the Interactive Visualization and Analysis of National Risk Standardized Mortality Rates of Sepsis in the US	Descriptive	Taiwan and United States of America	Diseases I Sepsis	National
**Luchetti, G., et al. (2017). Whistland: An Augmented Reality Crowd-Mapping System for Civil Protection and Emergency Management.** ***ISPRS International Journal of Geo-Information*****,** ***6*****(2), Article 41.**	Whistland	Descriptive	Italy	Crisis I Disaster I Natural Crisis I Emergencies	Local
**Marshall, B. D. L., et al. (2017). Development of a Statewide, Publicly Accessible Drug Overdose Surveillance and Information System.** ***American Journal of Public Health*****,** ***107*****(11), 1760-1763.**	Prevent Overdose Rhode Island (PORI)	User Study	United States of America	Diseases I Substance Abuse I Opioid	Regional
**Martinez, L. S., et al. (2019).** ***A Case Study in Belief Surveillance, Sentiment Analysis, and Identification of Informational Targets for E-Cigarettes Interventions.*** **SMSociety '19: Proceedings of the 10th International Conference on Social Media and Society, Toronto, ON.**	Social Media Analytics And Research Testbed (SMART) Dashboard	Descriptive	United States of America	Diseases I Substance Abuse I E-cigarettes	Local
**Meng, Y., et al. (2020). Lessons Learned in the Development of a Web-Based Surveillance Reporting System and Dashboard to Monitor Acute Febrile Illnesses in Guangdong and Yunnan Provinces, China, 2017-2019.** ***Health Security*****,** ***18*****(S1), 14-22.**	Epidemiologic Dynamic Data Collection (EDDC) Platform (modified and customized)	Descriptive	China	Infectious Diseases I Zika	Regional
**Mulero, R., et al. (2018). Towards Ambient Assisted Cities Using Linked Data and Data Analysis.** ***Journal of Ambient Intelligence and Humanized Computing*****,** ***9*****(5), 1573-1591.**	City4Age	Descriptive	Spain and Serbia	Health Related Services I Ageing Population I Mild Cognitive Impairments (MCI) and Frailty I Urban Health	Local
**Nascimento, B. S., et al. (2017).** ***A Flexible Architecture for Selection and Visualization of Information in Emergency Situations.*** **2016 IEEE International Conference on Systems, Man, and Cybernetics (SMC 2016), Budapest, Hungary.**	Emergency Dashboard	Descriptive	Brazil	Crisis I Emergencies	Local

**Pathirannehelage, S., et al. (2018). Uptake of a Dashboard Designed to Give Realtime Feedback to a Sentinel Network About Key Data Required for Influenza Vaccine Effectiveness Studies. *Studies in Health Technology and Informatics*, *247*, 161-165.**	My Practice Dashboard	User Study	United Kingdom	Infectious Diseases I Influenza I Vaccination	National
**Perez-Gonzalez, C. J., et al. (2019). Developing a Data Analytics Platform to Support Decision Making in Emergency and Security Management. *Expert Systems with Applications*, *120*, 167-184.**	Web platform with dashboards on social and economic indicators	Descriptive	Spain	Crisis I Emergencies	Regional
**Pike, I., et al. (2017). The Canadian Atlas of Child and Youth Injury: Mobilizing Injury Surveillance Data to Launch a National Knowledge Translation Tool. *International Journal of Environmental Research and Public Health*, *14*(9), 982, Article 982.**	Canadian Atlas of Child and Youth Injury Prevention	User Study	Canada	Diseases I Injuries	National
**Poy, A., et al. (2017). Monitoring Results in Routine Immunization: Development of Routine Immunization Dashboard in Selected African Countries in the Context of the Polio Eradication Endgame Strategic Plan. *Journal of Infectious Diseases*, *216*, 226-236.**	Dashboard on Routine Immunization	Descriptive	Congo, United States of America, Switzerland	Infectious Diseases I Polio	National
**Rees, E. E., et al. (2011). Advancements in Web-Database Applications for Rabies Surveillance. *International Journal of Health Geographics*, *10*, Article 48.**	RageDB	Descriptive	Canada	Infectious Diseases I Spread of a Pandemic I Rabies	Regional
**Rees, K. (2010). *Periscopic Visualizes Symptomatology of Pandemic: Vast 2010 Mini Challenge 2 Award: Effective Visualization of Symptoms.* 2010 IEEE Symposium on Visual Analytics Science and Technology, Salt Lake City, UT.**	Software and visualization methods on the spread of a pandemic	Descriptive	United States of America	Infectious Diseases I Spread of a Pandemic	International
**Robertson, H., et al. (2017). A Spatial Dashboard for Alzheimer's Disease in New South Wales. In A. Ryan, L. K. Schaper, & S. Whetton (Eds.), *Integrating and Connecting Care* (Vol. 239, pp. 126-132). Ios Press.**	Spatial Dashboard for Alzheimer's Disease	Descriptive	Australia	Health Related Services I Ageing Population I Dementia	Regional
**Ryan, K., et al. (2016). Development of an Obesity Prevention Dashboard for Wisconsin. *Wisconsin Medical Journal*, *115*(5), 224-227.**	Obesity Prevention Dashboard	Descriptive	United States of America	Diseases I Obesity	Regional
**Saha, S., et al. (2018). An Analytics Dashboard Visualization for Flood Decision Support System. *Journal of Visualisation*, *21*(2), 295–307.**	Analytics dashboard visualization for a flood decision support system	User Study	India	Crisis I Disaster	Regional
**Savini, L., et al. (2018). A Web Geographic Information System to Share Data and Explorative Analysis Tools: The Application to West Nile Disease in the Mediterranean Basin. *PLOS ONE*, *13*(6), Article e0196429.**	Disease Monitoring Dashboard (DMD)	Descriptive	Italy	Infectious Diseases I West Nile Virus	International
**Senyoni, W. F., et al. (2019). An Institutional Perspective on the Adoption of Open Dashboard for Health**	Open Dashboard	User Study	Norway, Tanzania	Health Related Services	National

**Information Systems in Tanzania. In P. Nielsen & H. C. Kimaro (Eds.), *Information and Communication Technologies for Development: Strengthening Southern-Driven Cooperation as a Catalyst for Ict4d, Pt I* (Vol. 551, pp. 272-283). Springer-Verlag Berlin.**					
**Singh, S. K. (2017). Conceptual Framework of a Cloud-Based Decision Support System for Arsenic Health Risk Assessment. *Environment Systems and Decisions*, *37*(4), 435-450.**	Global Arsenic Occurrence Dashboard (GAOD)	Descriptive	United States of America	Diseases I Pollution I Arsenic	International
**Tegtmeyer, R., et al. (2012). *Tracing and Responding to Foodborne Illness.* Proceedings of the 30th ACM International Conference on Design of Communication, Seattle, Washington, USA.**	Online system to track foodborne illnesses	Descriptive	United States of America	Diseases I Foodborne	National
**ter Waarbeek, H., et al. (2011). Strengthening Infectious Disease Surveillance in a Dutch-German Crossborder Area Using a Real-Time Information Exchange System. *Journal of business continuity & emergency planning*, *5*(2), 173-184.**	Crossborder Dashboard	Descriptive	The Netherlands, Germany, United Kingdom	Infectious Diseases	Regional
**Thomas, M., et al. (2016). The Role of Participatory Communication in Tracking Unreported Reproductive Tract Issues in Marginalized Communities. *Information Technology for Development*, *22*(1), 117–133.**	K-unit Health Information Dashboard (KHID)	Descriptive	United States of America, India	Diseases I Reproductive Tract Infections	Local
**Thomas, M. A., et al. (2012). Mitigating Gaps in Reproductive Health Reporting in Outlier Communities of Kerala, India-a Mobile Phone-Based Health Information System. *Health Policy and Technology*, *1*(2), 69-76.**	K-unit Health Information Dashboard (KHID)	Descriptive	United States of America, India	Health Related Services	Local
**Thorve, S., et al. (2018). EpiViewer: An Epidemiological Application for Exploring Time Series Data. *BMC Bioinformatics*, *19*(1), 449, Article 449.**	EpiViewer	User Study	United States of America	Infectious Diseases	International
**Tom-Aba, D., et al. (2015). Innovative Technological Approach to Ebola Virus Disease Outbreak Response in Nigeria Using the Open Data Kit and Form Hub Technology. *PLOS ONE*, *10*(6), Article e0131000.**	"Dashboard Technology"	Descriptive	Nigeria	Infectious Diseases I Ebola	National
**Urosevic, V., et al. (2017). *An Interactive Environment for Managing Detected Data Towards Geriatric Prevention.* 2017 IEEE 3rd International Forum on Research and Technologies for Society and Industry (RTSI), Modena, Italy.**	Individual Monitoring Dashboards (IMDs)	Descriptive	Serbia, Italy, Greece	Health Related Services I Ageing Population	Local
**van Ginkel, K. C. H., et al. (2018). Urban Water Security Dashboard: Systems Approach to Characterizing the Water Security of Cities [Article]. *Journal of Water Resources Planning and Management*, *144*(12), Article 04018075.**	Urban Water Security Dashboard	Descriptive	The Netherlands, Singapore	Diseases I Waterborne	Local

**Vila, R. A., et al. (2018).** ***The Design and Use of Dashboards for Driving Decision-Making in the Public Sector*** **Proceedings of the 11th International Conference on Theory and Practice of Electronic Governance, New York.**	Dashboards for decision-making in the public sector	Descriptive	Argentina	Health Related Services	Local
**Wahi, M. M., et al. (2019). Visualizing Infection Surveillance Data for Policymaking Using Open Source Dashboarding.** ***Applied Clinical Informatics*****,** ***10*****(3), 534-542.**	CAUTI SURVEILLANCE	Descriptive	United States of America	Diseases I Health Care associated Infections I Catheter-associated Urinary Tract Infections	Local
**Waye, K. M., et al. (2018). Action-Focused, Plain Language Communication for Overdose Prevention: A Qualitative Analysis of Rhode Island's Overdose Surveillance and Information Dashboard.** ***International Journal of Drug Policy*****,** ***62*****, 86-93.**	Prevent Overdose Rhode Island (PORI)	User Study	United States of America	Diseases I Substance Abuse I Opioids	Regional
**Wissel, B. D., et al. (2020). An Interactive Online Dashboard for Tracking COVID-19 in U.S. Counties, Cities, and States in Real Time.** ***Journal of the American Medical Informatics Association, 27*****(7), 1121-1125.**	COVID-19 Watcher Dashboard	Descriptive	United States of America	Infectious Diseases I Corona	National
**Zheng, L., et al. (2013). Data Mining Meets the Needs of Disaster Information Management.** ***IEEE Transactions on Human-Machine Systems*****,** ***43*****(5), 451-464.**	Dynamic Dashboard	User Study	United States of America	Crisis I Disaster I Hurricanes	Regional
**Zheng, L., et al. (2010).** ***Using Data Mining Techniques to Address Critical Information Exchange Needs in Disaster Affected Public-Private Networks.*** **Proceedings of the 16th ACM SIGKDD International Conference on Knowledge Discovery and Data Mining, Washington, DC.**	Dynamic Dashboard	User Study	United States of America	Crisis I Disaster I Hurricanes	Regional
**Zhu, Z., et al. (2017). Interactive Data Visualization to Understand Data Better: Case Studies in Healthcare System. In** ***Decision Management: Concepts, Methodologies, Tools, and Applications*** **(Vol. 1-4, pp. 27-36). IGI Global.**	Data Visualization in the Health System	Descriptive	Singapore	Health Related Services	Regional
