# Supplementary material for: Digital dashboards visualizing public health data: a systematic review
Source: Front Public Health. 2023 May 4;11:999958. doi: 10.3389/fpubh.2023.999958 (PMC10192578; doi:10.3389/fpubh.2023.999958)
Supplement: Supplementary file 5 [file Data_Sheet_5.PDF]

Appendix E

**Author (Publication Year)**					**Visualization Options**								
	Maps	*Symbols*	*Icons*	*Pop-ups*	*Bar Charts*	*Pie Charts*	*Line Graphs*	*Tables*	*Timeline*	*Tree Maps*	*Word Clouds*	*Sub-Sections*	*Use of Colour*
**Al Manir, M. S., et al. (2018). A Surveillance Infrastructure for Malaria Analytics: Provisioning Data Access and Preservation of Interoperability. *JMIR Public Health And Surveillance*, *4*(2), Article e10218.**													
**Alvarez, V. C., et al. (2019). Visualization of Health Data. In J. C. W. Lin, I. H. Ting, K. Wang, & T. Tang (Eds.), *Multidisciplinary Social Networks Research, 6th International Conference, MISNC 2019, Wenzhou, China, August 26–28, 2019, Revised Selected Papers* (Vol. 1131 CCIS, pp. 118-130): Springer.**	X			X	X		X						
**Avvenuti, M., et al. (2018). CrisMap: A Big Data Crisis Mapping System Based on Damage Detection and Geoparsing. *Information Systems Frontiers*, *20*(5), 993–1011.**	X										X		
**Basdere, M., et al. (2019). Safe: A Comprehensive Data Visualization System. *INFORMS Journal on Applied Analytics*, *49*(4), 249-261.**	X			X	X								X
**Benson, A. L., et al. (2010). *Adaptive Development of a Common Operating Environment for Crisis Response and Management.* ISCRAM 2010 – 7th International Conference on Information Systems for Crisis Response and Management: Defining Crisis Management 3.0, Proceedings, Seattle, WA: Information Systems for Crisis Response and Management, ISCRAM.**	X												
**Bernard, J., et al. (2019). Using Dashboard Networks to Visualize Multiple Patient Histories: A Design Study on Post-Operative Prostate Cancer. *IEEE Transactions on Visualization and Computer Graphics*, *25*(3), 1615-1628.**	X				X	X							X
**Bhardwaj, S., et al. (2014). Elimination of Mother-to-Child Transmission of HIV in South Africa: Rapid Scale-up Using Quality Improvement. *South African Medical Journal*, *104*(3), 239-243.**													X

Braa, J., et al. (2017). Health Information Systems in Indonesia: Understanding and Addressing Complexity. In M. S. Islam, F. Wahid, J. E. Priyatma, J. Choudrie, & J. M. Bass (Eds.), (Vol. 504, pp. 59-70): Springer New York LLC.													
Brownson, R. C., et al. (2015). Applying A Mixed-Methods Evaluation to Healthy Kids, Healthy Communities. *Journal of Public Health Management and Practice*, *21*, 16-26.													
Campbell, T. C., et al. (2014). Development of the Respiratory Disease Dashboard for the Identification of New and Emerging Respiratory Pathogens. *Johns Hopkins APL Technical Digest (Applied Physics Laboratory)*, *32*(4), 726-734.	X					X							X
Carmichael, J. M., et al. (2017). Leveraging Electronic Medical Record Data for Population Health Management in the Veterans Health Administration: Successes and Lessons Learned. *American Journal of Health-System Pharmacy*, *74*(18), 1447-1459.		X											X
Choudhary, V., et al. (2020). *AirQ: A Smart IOT Platform for Air Quality Monitoring.* 2020 IEEE 17th Annual Consumer Communications & Networking Conference (CCNC), Las Vegas, NV.	X												
Concannon, D., et al. (2019). Developing a Data Dashboard Framework for Population Health Surveillance: Widening Access to Clinical Trial Findings. *JMIR Formative Research*, *3*(2), Article e11342.	X		X	X	X	X				X			X
Devi, L. N., et al. (2018). *Live Demonstration on Smart Water Quality Monitoring System Using Wireless Sensor Networks.* 2018 IEEE SENSORS, New Delhi, India.	X												
Dong, E., et al. (2020). An Interactive Web-Based Dashboard to Track COVID-19 in Real Time. *The Lancet. Infectious Diseases*, *20*(5), 533-534.													
Erraguntla, M., et al. (2012). *Open Source Text Based Biovigilance.* Proceedings of the 2012 International Conference on Artificial Intelligence (ICAI 2012, Vol. 1), Las Vegas, NV.									X				
Estuar, M. R. E., et al. (2016). *The Challenge of Continuous User Participation in eBayanihan: Digitizing Humanitarian Action in a Nationwide Web Mobile Participatory Disaster Management System.* 2016 3rd International Conference on Information and	X												

Communication Technologies for Disaster Management (ICT-DM), Vienna, Austria.													
Federico, L., et al. (2016). *SINSE+: A Software for the Acquisition and Analysis of Open Data in Health and Social Area* 24th Italian Symposium on Advanced Database Systems (SEBD 2016), Ugento, Lecce, Italy.	X						X						
Gourevitch, M. N., et al. (2019). City-Level Measures of Health, Health Determinants, and Equity to Foster Population Health Improvement: The City Health Dashboard. *American Journal of Public Health*, *109*(4), 585-592.	X							X					
Hamoy, G. L., et al. (2016). Real-Time Regular Routine Reporting for Health (R4health): Lessons from the Implementation of a Large Scale Mobile Health System for Routine Health Services in the Philippines. *Acta Medica Philippina*, *50*(4), 280-294.		X			X								
Harris, J. K., et al. (2018). Evaluating the Implementation of a Twitter-Based Foodborne Illness Reporting Tool in the City of St. Louis Department of Health. *International Journal of Environmental Research and Public Health*, *15*(5), Article 833.													
Hoare, G., et al. (2010). Developing H1N1 Hospital Surge "Dashboard" Indicators: A Demonstration. ISCRAM 2010 – 7th International Conference on Information Systems for Crisis Response and Management: Defining Crisis Management 3.0.													
Homsuwan, P., et al. (2018). Visualization Development of Health Data Reporting with Business Intelligence Techniques. *Journal of the Medical Association of Thailand*, *101*(6), 49-54.	X				X	X							X
Husain, S. S., et al. (2015). SOCR Data Dashboard: An Integrated Big Data Archive Mashing Medicare, Labor, Census and Econometric Information. *Journal of Big Data*, *2*(1), Article 13.	X				X	X		X					
Husain, W., et al. (2016). M-DENGUE: Utilizing Crowdsourcing and Teleconsultation for Location-Based Dengue Monitoring and Reporting System. *Jurnal Teknologi*, *78*(9-3), 89-95.	X				X	X							
Jamil, J. M., et al. (2016). An Innovative Data Mining and Dashboard System for Monitoring of Malaysian Dengue Trends. *Journal of Telecommunication, Electronic and Computer Engineering*, *8*(10), 9-12.					X	X	X	X					

Jinpon, P., et al. (2017). Integrated Information Visualization to Support Decision Making for Health Promotion in Chonburi, Thailand. *Walailak Journal of Science and Technology*, *16*(8), 551-560.					X		X	X					
Jinpon, P., et al. (2017). Integrated Information Visualization to Support Decision-Making in Order to Strengthen Communities: Design and Usability Evaluation. *Informatics for Health & Social Care*, *42*(4), 335-348.	X				X	X		X					
Kamadjeu, R., et al. (2017). Designing and Implementing an Electronic Dashboard for Disease Outbreaks Response - Case Study of the 2013-2014 Somalia Polio Outbreak Response Dashboard. *The Pan African medical journal*, *27*.	X				X	X		X					
Kostkova, P. (2013). *A Roadmap to Integrated Digital Public Health Surveillance: The Vision and the Challenges.* WWW '13 Companion Proceedings of the 22nd International Conference on World Wide Web, Rio de Janeiro, Brazil.	X												
Kostkova, P., et al. (2014). *Integration and Visualization Public Health Dashboard: The Medi+Board Pilot Project.* WWW '14 Companion: Proceedings of the 23rd International Conference on World Wide Web, Seoul, Korea.	X				X	X		X	X				
Lee, M. T., et al. (2020). Web-Based Dashboard for the Interactive Visualization and Analysis of National Risk-Standardized Mortality Rates of Sepsis in the US. *Journal of Medical Systems*, *44*(2), Article 54.	X							X					X
Luchetti, G., et al. (2017). Whistland: An Augmented Reality Crowd-Mapping System for Civil Protection and Emergency Management. *ISPRS International Journal of Geo-Information*, *6*(2), Article 41.	X							X					
Marshall, B. D. L., et al. (2017). Development of a Statewide, Publicly Accessible Drug Overdose Surveillance and Information System. *American Journal of Public Health*, *107*(11), 1760-1763.	X			X									
Martinez, L. S., et al. (2019). *A Case Study in Belief Surveillance, Sentiment Analysis, and Identification of Informational Targets for E-Cigarettes Interventions.* SMSociety '19: Proceedings of the 10th International Conference on Social Media and Society, Toronto, ON.											X		

Meng, Y., et al. (2020). Lessons Learned in the Development of a Web-Based Surveillance Reporting System and Dashboard to Monitor Acute Febrile Illnesses in Guangdong and Yunnan Provinces, China, 2017-2019. *Health Security*, *18*(S1), 14-22.	X							X					
Mulero, R., et al. (2018). Towards Ambient Assisted Cities Using Linked Data and Data Analysis. *Journal of Ambient Intelligence and Humanized Computing*, *9*(5), 1573-1591.					X								
Nascimento, B. S., et al. (2017). *A Flexible Architecture for Selection and Visualization of Information in Emergency Situations.* 2016 IEEE International Conference on Systems, Man, and Cybernetics (SMC 2016), Budapest, Hungary.	X				X	X		X		X			X
Pathirannehelage, S., et al. (2018). Uptake of a Dashboard Designed to Give Realtime Feedback to a Sentinel Network About Key Data Required for Influenza Vaccine Effectiveness Studies. *Studies in Health Technology and Informatics*, *247*, 161-165.				X	X			X					X
Perez-Gonzalez, C. J., et al. (2019). Developing a Data Analytics Platform to Support Decision Making in Emergency and Security Management. *Expert Systems with Applications*, *120*, 167-184.	X							X					
Pike, I., et al. (2017). The Canadian Atlas of Child and Youth Injury: Mobilizing Injury Surveillance Data to Launch a National Knowledge Translation Tool. *International Journal of Environmental Research and Public Health*, *14*(9), 982, Article 982.	X			X	X				X			X	X
Poy, A., et al. (2017). Monitoring Results in Routine Immunization: Development of Routine Immunization Dashboard in Selected African Countries in the Context of the Polio Eradication Endgame Strategic Plan. *Journal of Infectious Diseases*, *216*, 226-236.													
Rees, E. E., et al. (2011). Advancements in Web-Database Applications for Rabies Surveillance. *International Journal of Health Geographics*, *10*, Article 48.	X	X				X		X				X	
Rees, K. (2010). *Periscopic Visualizes Symptomatology of Pandemic: Vast 2010 Mini Challenge 2 Award: Effective Visualization of Symptoms.* 2010 IEEE Symposium on Visual Analytics Science and Technology, Salt Lake City, UT.	X	X					X						X

Robertson, H., et al. (2017). A Spatial Dashboard for Alzheimer's Disease in New South Wales. In A. Ryan, L. K. Schaper, & S. Whetton (Eds.), *Integrating and Connecting Care* (Vol. 239, pp. 126-132). Ios Press.	X											X	X
Ryan, K., et al. (2016). Development of an Obesity Prevention Dashboard for Wisconsin. *Wisconsin Medical Journal*, *115*(5), 224-227.					X								
Saha, S., et al. (2018). An Analytics Dashboard Visualization for Flood Decision Support System. *Journal of Visualisation*, *21*(2), 295–307.	X			X									X
Savini, L., et al. (2018). A Web Geographic Information System to Share Data and Explorative Analysis Tools: The Application to West Nile Disease in the Mediterranean Basin. *PLOS ONE*, *13*(6), Article e0196429.	X	X				X		X					X
Senyoni, W. F., et al. (2019). An Institutional Perspective on the Adoption of Open Dashboard for Health Information Systems in Tanzania. In P. Nielsen & H. C. Kimaro (Eds.), *Information and Communication Technologies for Development: Strengthening Southern-Driven Cooperation as a Catalyst for Ict4d, Pt I* (Vol. 551, pp. 272-283). Springer-Verlag Berlin.												X	
Singh, S. K. (2017). Conceptual Framework of a Cloud-Based Decision Support System for Arsenic Health Risk Assessment. *Environment Systems and Decisions*, *37*(4), 435-450.	X					X		X					
Tegtmeyer, R., et al. (2012). *Tracing and Responding to Foodborne Illness.* Proceedings of the 30th ACM International Conference on Design of Communication, Seattle, Washington, USA.	X										X		
ter Waarbeek, H., et al. (2011). Strengthening Infectious Disease Surveillance in a Dutch-German Crossborder Area Using a Real-Time Information Exchange System. *Journal of business continuity & emergency planning*, *5*(2), 173-184.	X							X					
Thomas, M., et al. (2016). The Role of Participatory Communication in Tracking Unreported Reproductive Tract Issues in Marginalized Communities. *Information Technology for Development*, *22*(1), 117–133.					X	X		X					
Thomas, M. A., et al. (2012). Mitigating Gaps in Reproductive Health Reporting in Outlier Communities of Kerala, India-a Mobile Phone-Based Health Information System. *Health Policy and Technology*,					X	X		X					

1(2), 69-76.													
Thorve, S., et al. (2018). EpiViewer: An Epidemiological Application for Exploring Time Series Data. *BMC Bioinformatics*, *19*(1), 449, Article 449.				X	X		X					X	X
Tom-Aba, D., et al. (2015). Innovative Technological Approach to Ebola Virus Disease Outbreak Response in Nigeria Using the Open Data Kit and Form Hub Technology. *PLOS ONE*, *10*(6), Article e0131000.	X												
Urosevic, V., et al. (2017). *An Interactive Environment for Managing Detected Data Towards Geriatric Prevention.* 2017 IEEE 3rd International Forum on Research and Technologies for Society and Industry (RTSI), Modena, Italy.			X	X	X				X			X	
van Ginkel, K. C. H., et al. (2018). Urban Water Security Dashboard: Systems Approach to Characterizing the Water Security of Cities [Article]. *Journal of Water Resources Planning and Management*, *144*(12), Article 04018075.													
Vila, R. A., et al. (2018). *The Design and Use of Dashboards for Driving Decision-Making in the Public Sector* Proceedings of the 11th International Conference on Theory and Practice of Electronic Governance, New York.	X			X									X
Wahi, M. M., et al. (2019). Visualizing Infection Surveillance Data for Policymaking Using Open Source Dashboarding. *Applied Clinical Informatics*, *10*(3), 534-542.					X							X	
Waye, K. M., et al. (2018). Action-Focused, Plain Language Communication for Overdose Prevention: A Qualitative Analysis of Rhode Island's Overdose Surveillance and Information Dashboard. *International Journal of Drug Policy*, *62*, 86-93.	X		X										
Wissel, B. D., et al. (2020). An Interactive Online Dashboard for Tracking COVID-19 in U.S. Counties, Cities, and States in Real Time. *Journal of the American Medical Informatics Association*, *27*(7), 1121-1125.	X							X					
Zheng, L., et al. (2013). Data Mining Meets the Needs of Disaster Information Management. *IEEE Transactions on Human-Machine Systems*, *43*(5), 451-464.	X		X										
Zheng, L., et al. (2010). *Using Data Mining Techniques to Address Critical Information Exchange Needs in*			X					X				X	X

Disaster Affected Public-Private Networks. Proceedings of the 16th ACM SIGKDD International Conference on Knowledge Discovery and Data Mining, Washington, DC.													
Zhu, Z., et al. (2017). Interactive Data Visualization to Understand Data Better: Case Studies in Healthcare System. In *Decision Management: Concepts, Methodologies, Tools, and Applications* (Vol. 1-4, pp. 27-36). IGI Global.	x		x	x	x							x	x
