# Supplementary material for: Digital dashboards visualizing public health data: a systematic review
Source: Front Public Health. 2023 May 4;11:999958. doi: 10.3389/fpubh.2023.999958 (PMC10192578; doi:10.3389/fpubh.2023.999958)
Supplement: Supplementary file 9 [file Data_Sheet_9.PDF]

Appendix I

Author (Publication Year)	Target Group	Study Participants	Needs
**Bernard, J., et al. (2019). Using Dashboard Networks to Visualize Multiple Patient Histories: A Design Study on Post-Operative Prostate Cancer. *IEEE Transactions on Visualization and Computer Graphics*, *25*(3), 1615-1628.**	Medicine I Health workers<br>public I Non-experts<br>Others I Visualisation Experts	Medicine I health workers<br>public I non-experts<br>others I visualisation experts	Intuitiveness I Segregation of Data I Colour Use
**Concannon, D., et al. (2019). Developing a Data Dashboard Framework for Population Health Surveillance: Widening Access to Clinical Trial Findings. *JMIR Formative Research*, *3*(2), Article e11342.**	Research I Researchers<br>Medicine I Health Workers<br>Public I Community Members	Research I Researchers<br>Medicine I Health Workers<br>Public I Community Members	Demands Varied Between User Groups: Understandability I Use of Terms I Design I Chart Series
**Estuar, M. R. E., et al. (2016). *The Challenge of Continuous User Participation in eBayanihan: Digitizing Humanitarian Action in a Nationwide Web Mobile Participatory Disaster Management System.* 2016 3rd International Conference on Information and Communication Technologies for Disaster Management (ICT-DM), Vienna, Austria.**	Public I Citizens<br>Policy I Local<br>Policy I National	Not Specified	Engagement I Post Messages<br>Engagement I User Rankings<br>Engagement I Notifications
**Gourevitch, M. N., et al. (2019). City-Level Measures of Health, Health Determinants, and Equity to Foster Population Health Improvement: The City Health Dashboard. *American Journal of Public Health*, *109*(4), 585-592.**	Public I Specific I Stakeholders	Public I Specific I Stakeholders	Data I High Frequency of Data Actualisation
**Hamoy, G. L., et al. (2016). Real-Time Regular Routine Reporting for Health (R4health): Lessons from the Implementation of a Large Scale Mobile Health System for Routine Health Services in the Philippines. *Acta Medica Philippina*, *50*(4), 280-294.**	Medicine I Health Workers	Medicine I Health Workers	Practicability I Compatibility With Workflow<br>Practicability I Facilitation of Work Tasks<br>Practicability I Use in Rural Areas<br>Data I Timely and Accurate Data<br>Literacy I Technical Knowledge<br>Engagement I Hotline
**Harris, J. K., et al. (2018). Evaluating the Implementation of a Twitter-Based Foodborne Illness Reporting Tool in the City of St. Louis Department of Health. *International Journal of Environmental Research and Public Health*, *15*(5), Article 833.**	Public Health<br>Research I Epidemiologists<br>Policy	Public Health<br>Research I Epidemiologists<br>Policy	Practicability I Adaptability<br>Practicability I Compatibility With Workflow I No Extra Work<br>Accessibility I Access Technology<br>Engagement I Social Media
**Homsuwan, P., et al. (2018). Visualization Development of Health Data Reporting with Business Intelligence Techniques. *Journal of the Medical Association of Thailand*, *101*(6), 49-54.**	Medicine I Health Workers	Medicine I Health Workers	Accessibility I Access Technology<br>Practicability I Facilitation of Work Tasks
**Jinpon, P., et al. (2017). Integrated Information Visualization to Support Decision Making for Health Promotion in Chonburi, Thailand. *Walailak Journal of Science and Technology*, *16*(8), 551-560.**	Medicine I Health Workers I Nurses<br>Medicine I Health Workers I Administrators<br>Medicine I Practitioners<br>Medicine I Other Professionals	Medicine I Health Workers I Nurses<br>Medicine I Health Workers I Administrators<br>Medicine I Practitioners<br>Medicine I Other Professionals	Literacy I Learnability of the System<br>Time I Fast Provision of Results<br>System Efficiency<br>Design I Availability of Different Formats I Graphs

**Jinpon, P., et al. (2017). Integrated Information Visualization to Support Decision-Making in Order to Strengthen Communities: Design and Usability Evaluation.** ***Informatics for Health & Social Care*****,** ***42*****(4), 335-348.**	Policy I Local I Local Council<br>Policy I Local I Administration<br>Medical I Health Workers I Administrators<br>Public I General	Policy I Local I Local Council<br>Policy I Local I Administration<br>Medical I Health Workers I Administrators<br>Public I General	Preferences Regarding the Tool Varied Between User Groups:<br>Practicability I Facilitation of Work Tasks<br>Data I Easy Access I Decision-Making<br>Design I Trend Animation
**Marshall, B. D. L., et al. (2017). Development of a Statewide, Publicly Accessible Drug Overdose Surveillance and Information System.** ***American Journal of Public Health*****,** ***107*****(11), 1760-1763.**	Public I General	Public I Specific I Stakeholders	Design I Maps
**Pathirannehelage, S., et al. (2018). Uptake of a Dashboard Designed to Give Realtime Feedback to a Sentinel Network About Key Data Required for Influenza Vaccine Effectiveness Studies.** ***Studies in Health Technology and Informatics*****,** ***247*****, 161-165.**	Research I Researchers	Research I Researchers	Accessibility I Network<br>Data I Easy Loading<br>Data I Summary<br>Availability I Available at any Time<br>Support in Case of Uncertainty<br>Design I Layout<br>Data I Download
**Pike, I., et al. (2017). The Canadian Atlas of Child and Youth Injury: Mobilizing Injury Surveillance Data to Launch a National Knowledge Translation Tool.** ***International Journal of Environmental Research and Public Health*****,** ***14*****(9), 982, Article 982.**	Public Health I Other Experts<br>Policy I Local<br>Policy I National	Public Health I Other Experts<br>Policy I Local<br>Policy I National	Intuitiveness I Interface<br>Support in Case of Uncertainty<br>Customization
**Saha, S., et al. (2018). An Analytics Dashboard Visualization for Flood Decision Support System.** ***Journal of Visualisation*****,** ***21*****(2), 295–307.**	Policy I National I Government Officials	Policy I National I Government Officials	Time I Real-Time Monitoring<br>Design I Interactivity
**Senyoni, W. F., et al. (2019). An Institutional Perspective on the Adoption of Open Dashboard for Health Information Systems in Tanzania. In P. Nielsen & H. C. Kimaro (Eds.),** ***Information and Communication Technologies for Development: Strengthening Southern-Driven Cooperation as a Catalyst for Ict4d, Pt I*** **(Vol. 551, pp. 272-283). Springer-Verlag Berlin.**	Public Health I Other Experts	Public Health I Other Experts	x
**Thorve, S., et al. (2018). EpiViewer: An Epidemiological Application for Exploring Time Series Data.** ***BMC Bioinformatics*****,** ***19*****(1), 449, Article 449.**	Research I Researchers	Research I Researchers<br>Research I Students	Data I Upload<br>Data I Grouping<br>Easy Learnability<br>Engagement I Feedback Messages
**Waye, K. M., et al. (2018). Action-Focused, Plain Language Communication for Overdose Prevention: A Qualitative Analysis of Rhode Island's Overdose Surveillance and Information Dashboard.** ***International Journal of Drug Policy*****,** ***62*****, 86-93.**	Public I Specific I People who use Drugs	Public I Specific I People who use Drugs	Language I Target Group Specific<br>Ease of Use<br>Design I Interactivity
**Zheng, L., et al. (2013). Data Mining Meets the Needs of Disaster Information Management.** ***IEEE Transactions on Human-Machine Systems*****,** ***43*****(5), 451-464.**	Economy I Public Companies<br>Economy I Private Companies<br>Public Health I Disaster Management	Economy I Public Companies<br>Economy I Private Companies<br>Public Health I Disaster Management	Data I Report Summaries<br>Ease of Use<br>Design I Images and Videos
**Zheng, L., et al. (2010).** ***Using Data Mining Techniques to Address Critical Information Exchange Needs in Disaster Affected Public-Private Networks.*** **Proceedings of the 16th ACM SIGKDD International Conference on Knowledge Discovery and Data Mining, Washington, DC.**	Economy I Companies<br>Policy I Local I Government Officials	Public and Private Sector Partners	Engagement I Messaging<br>Data I Reporting<br>Practicability I Situational Browsing
