# Supplementary material for: Digital dashboards visualizing public health data: a systematic review
Source: Front Public Health. 2023 May 4;11:999958. doi: 10.3389/fpubh.2023.999958 (PMC10192578; doi:10.3389/fpubh.2023.999958)
Supplement: Supplementary file 10 [file Data_Sheet_10.PDF]

Appendix J MMAT Results

Types of study design	Methodological quality criteria	Bernard et al. 2019	Concannon et al. 2019	Estuar et al. 2016	Gourevitch et al. 2019	Hamoy et al. 2016	Harris et al. 2018	Homsuwan et al. 2018	Jinpon et al. 2017a	Jinpon et al. 2017b	Marshall et al. 2017	Pathriannehelage et al. 2018	Pike et al. 2017	Saha et al. 2018	Senyoni et al. 2019	Thorve et al. 2018	Waye et al. 2018	Zheng et al. 2010	Zheng et al. 2013
**Screening questions (for all types)**	S1. Are there clear research questions?	?	X	X	X	X	X	X	X	X	X	X	X	X	√	X	√	√	√
	S1.1. Are there research objectives?	√	√	√	√	√	√	√	√	√	√	√	√	√	-	√	-	-	-
	S2. Do the collected data allow to address the research questions/objectives?	√	√	?	?	√	√	√	√	√	?	√	√	√	√	√	√	√	√
**1. Qualitative**	1.1. Is the qualitative approach appropriate to answer the research question?	√	√	-	√	√	√	-	-	-	-	-	√	?	√	√	√	√	-
	1.2. Are the qualitative data collection methods adequate to address the research question?	√	√	-	?	√	√	-	-	-	-	-	√	√	√	√	√	√	-
	1.3. Are the findings adequately derived from the data?	√	?	-	?	?	?	-	-	-	-	-	?	?	?	√	√	?	-
	1.4. Is the interpretation of results sufficiently substantiated by data?	√	√	-	X	√	√	-	-	-	-	-	?	?	?	√	√	?	-
	1.5. Is there coherence between qualitative data sources, collection, analysis and interpretation?	√	?	-	?	√	√	-	-	-	-	-	?	?	?	√	√	?	-
**2. Randomized controlled trials**		-	-	-	-	-	-	-	-	-	-	-	-	-	-	-	-	-	-
**3. Non-randomized studies**		-	-	-	-	-	-	-	-	-	-	-	-	-	-	-	-	-	-
**4. Quantitative descriptive**	4.1. Is the sampling strategy relevant to address the research question?	-	√	?	-	?	-	?	?	?	?	?	-	-	-	√	-	-	?

	4.2. Is the sample representative of the target population?	-	√	?	-	√	-	√	√	√	√	√	-	-	-	?	-	-	?
	4.3. Are the measurements appropriate?	-	?	?	-	√	-	?	√	?	?	?	-	-	-	√	-	-	?
	4.4. Is the risk of non-response bias low?	-	?	X	-	?	-	?	?	?	?	?	-	-	-	?	-	-	?
	4.5. Is the statistical analysis appropriate to answer the research question?	-	?	√	-	√	-	√	√	√	?	?	-	-	-	?	-	-	?
**5. Mixed methods**	5.1. Is there an adequate rationale for using a mixed methods design to address the research question?	-	?	-	-	?	-	-	-	-	-	-	-	-	-	√	-	-	-
	5.2. Are the different components of the study effectively integrated to answer the research question?	-	?	-	-	√	-	-	-	-	-	-	-	-	-	√	-	-	-
	5.3. Are the outputs of the integration of qualitative and quantitative components adequately interpreted?	-	?	-	-	?	-	-	-	-	-	-	-	-	-	?	-	-	-
	5.4. Are divergences and inconsistencies between quantitative and qualitative results adequately addressed?	-	?	-	-	X	-	-	-	-	-	-	-	-	-	?	-	-	-
	5.5. Do the different components of the study adhere to the quality criteria of each tradition of the methods involved?	-	?	-	-	√	-	-	-	-	-	-	-	-	-	√	-	-	-

Note: “√“ = *yes; „X“ = no; „?“ = cannot tell; “-“ = not applicable*
